# Membraneless organelles-based integrative analysis constructs an immune-related prognostic signature and identifies NRG1 as a novel methylation biomarker in colorectal cancer

**DOI:** 10.3389/fimmu.2025.1678096

**Published:** 2025-10-20

**Authors:** Jingsong Cheng, Nanting Chen, Qingyao Yin, Ziheng Zheng, Xue Chen, Xinyi Zhu, Yuanyuan Wan, Ningxi Wang, Siqi Luo, Chengxi Zhang, Guodong Liu, Weilong Chen, Rugang Luo

**Affiliations:** ^1^ Department of Medical Oncology, Sun Yat-sen University Cancer Center, Guangzhou, China; ^2^ The Second Clinical College, The Second Affiliated Hospital of Chongqing Medical University, Chongqing Medical University, Chongqing, China; ^3^ College of Basic Medicine, Chongqing Medical University, Chongqing, China; ^4^ Department of Hematology, The Second Affiliated Hospital of Chongqing Medical University, Chongqing, China; ^5^ Department of Gastrointestinal surgery, Chongqing Hospital of the First Affiliated Hospital of Guangzhou University of Chinese Medicine, Chongqing, China; ^6^ Department of Neurosurgery, The Second Affiliated Hospital of Chongqing Medical University, Chongqing, China; ^7^ Department of Oncology, The People's Hospital of Chongqing Liangping District, Chongqing, China; ^8^ Department of Neurosurgery, The People’s Hospital of Renhuai, Renhuai City, Guizhou, China

**Keywords:** colorectal cancer, Nrg1, liquid-liquid phase separation, prognosis, methylation, membraneless organelle

## Abstract

**Background:**

The dysfunction of membraneless organelles (MLOs) has been implicated in tumorigenesis and progression by aberrant liquid–liquid phase separation (LLPS). However, the role of MLOs in the prognosis and tumor immune microenvironment (TIME) of colorectal cancer (CRC) remains unclear.

**Method:**

We integrated transcriptomic data of MLO-related genes to identify distinct CRC subtypes and constructed a prognostic risk score termed MPRS. Then, we systematically demonstrated the characteristics of MPRS based on multi-omics analyses. We further assessed NRG1’s LLPS possibility, prognostic significance, and its correlation with methylation through comprehensive analysis and *in vitro* experiment.

**Results:**

A prognostic signature called MPRS associated with prognosis, tumor ecotypes, genomic alterations, TIME patterns, immunotherapy responses, chemotherapy sensitivity in CRC patients. NRG1, identified as the most important MPRS gene with high predicted LLPS propensity—was significantly downregulated in CRC tissues and correlated with prognosis. Promoter methylation was found to be a crucial mechanism underlying NRG1 downregulation, which could be rescued by 5-Aza-2-deoxycytidine (Aza) treatment. The qRT-PCR, IHC and Aza treatment were utilized for *in vitro* validation.

**Conclusion:**

Our integrated multi-omics analysis constructed the MPRS model to delineate CRC tumor ecology and identified NRG1 as a methylation biomarker with predicted phase-separation propensity, with potential therapeutic implications that warrant prospective validation.

## 1 Introduction

Colorectal cancer (CRC), a prevalent cancer type, ranks as the third leading cause of death worldwide, claiming over 600,000 lives each year (1, 2). Furthermore, the notable variability in patient outcomes and treatment responses restricts the universal applicability of standard therapies. Recent breakthroughs, such as the work by Federica Papaccio and colleagues, have illuminated the molecular landscapes of advanced CRC characterized by chromosomal instability (CIN) (3). Concurrently, emerging evidence highlights the critical role of stress granules (SGs) in CRC pathogenesis (4). Despite these advances, significant gaps remain in understanding how subcellular organizational dynamics link to tumor ecology and clinical outcomes, significantly hindering the development of effective treatment strategies. For instance, Neuregulin-1 (NRG1), a growth factor, has been shown to influence cell survival, proliferation, migration, and differentiation by activating the epidermal growth factor receptor (EGFR) and its downstream pathways (5, 6). In CRC, overactivation of EGFR is often associated with poorer prognosis (7, 8); however, existing studies have demonstrated that high NRG1 expression in CRC patients correlates with better prognosis (9–11). The emergence of such contradictions necessitates exploring novel role of NRG1 via multi-omics landscape and cellular models to elucidate CRC pathogenesis.

Liquid–liquid phase separation (LLPS) refers to a phenomenon in which a homogeneous liquid solution spontaneously separates into two distinct liquid phases, usually due to the presence of specific components or conditions (12). LLPS, as a novel concept elucidating the complex organizational rules of living cells, has been shown to participate in forming various membraneless organelles (MLOs), including stress granules, heterochromatin, and transport channels within the nuclear pore complex (13–15). MLOs, also referred to as liquid condensates or liquid droplets, play essential roles in diverse biological processes and are vital for human well-being (16, 17). Recent studies indicate that abnormal MLOs are linked to various cancers by interfering with tumor suppression, disrupting normal signaling pathways, hyperactivating oncogenes, replication stress or impacting protein quality control mechanisms (18, 19). For the development of CRC, many tumorigenesis in relation to abnormal MLOs and LLPS were found recently. The Gαi2 mutant was associated with axin2 MLO dysfunction, affecting β-catenin degradation (20). APC mutants can impair phase separation capability, resulting in Wnt pathway hyperactivation (21, 22). Therefore, exploring the role of MLOs in CRC represents a promising avenue in the field of oncology treatment. These studies will significantly improve the understanding of tumor pathogenesis, aid in prognosis prediction, and support personalized treatment selection.

In our study, we identified distinct subtypes according to the MLO-related transcriptional profiles of CRC patient. The MLO-related prognostic risk score (MPRS) was developed as a novel prognostic signature, demonstrating strong predictive capabilities for prognosis, tumor immune microenvironment (TIME) patterns, immune checkpoint blockade (ICB) response, and chemotherapy sensitivity selection. Ultimately, we identified NRG1 as the MPRS component with the strongest LLPS potential and a potential link to KRAS mutation, which prompted us to further investigate and validate it as the most critical MLO-related gene (MRG) in CRC for its biomarker properties. We further established NRG1’s significant prognostic value in CRC and observed a specific downregulation of its expression in CRC tissues. Intriguingly, we found that NRG1 silencing is mediated by promoter methylation and can be rescued by treatment with 5-Aza-2-deoxycytidine (Aza). These findings suggest that NRG1 may serve as a promising prognostic biomarker and therapeutic for target CRC.

## 2 Methods

### 2.1 Study design workflow

**Figure 1:**
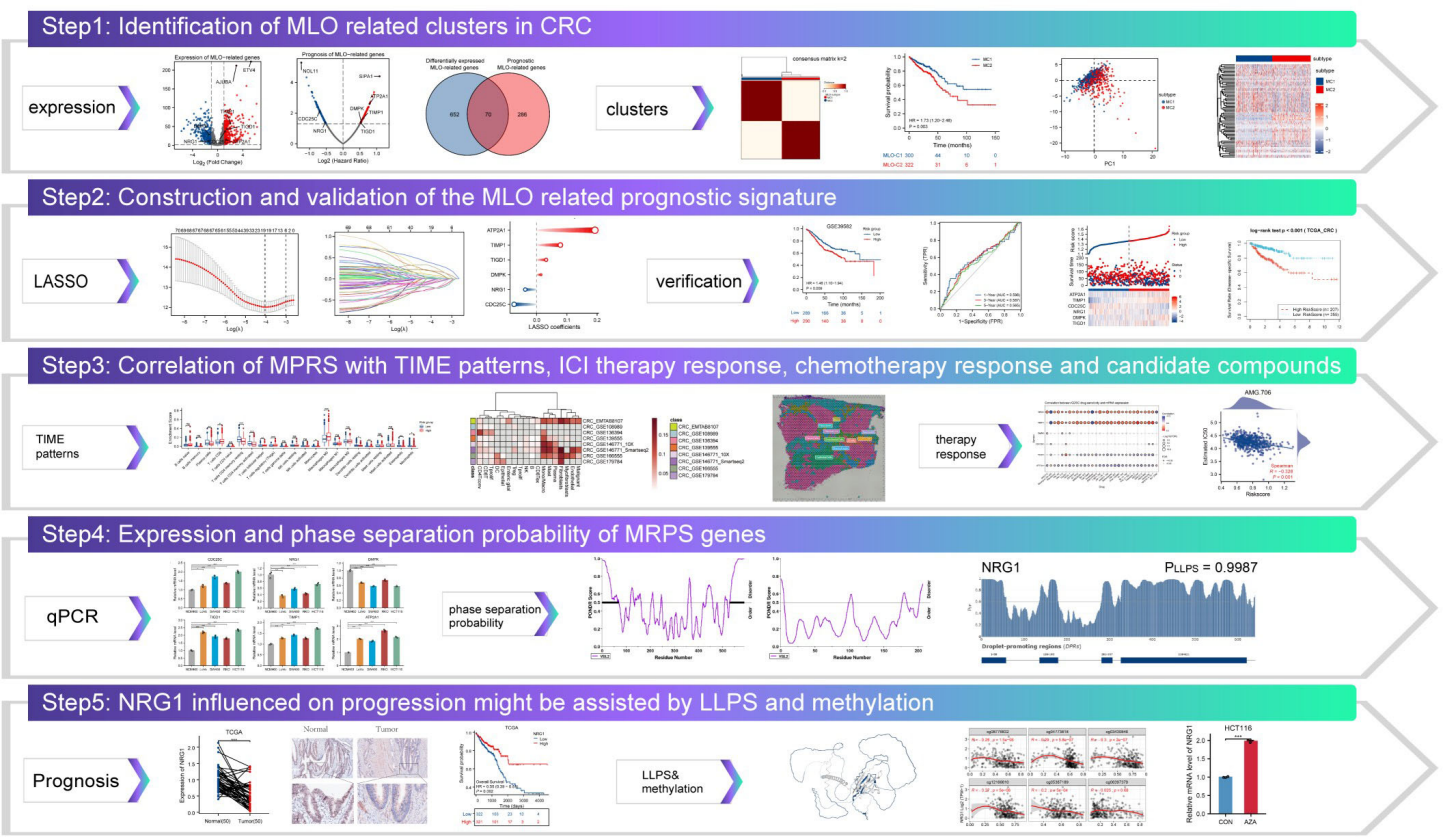
The integrative research framework.

The integrative research framework is schematically presented in **Figure 1**. Initial transcriptomic integration of MRGs enabled identification of differentially expressed prognostic genes in CRC versus normal tissues. Subsequent consensus clustering stratified CRC patients into molecularly defined MLO subtypes. We developed a robust prognostic signature called MPRS using LASSO regression, which was comprehensively validated across multiple domains: clinical outcomes, pathological characteristics, tumor ecotypes, genomic instability, TIME profiles, immunotherapy efficacy, and chemosensitivity. Further investigation focused on NRG1, which was a pivotal prognostic determinant identified through MPRS with high LLPS propensity. Experimental validation confirmed NRG1 downregulation in CRC, its association with adverse prognosis, and epigenetic silencing via promoter hypermethylation.

### 2.2 Data curation and harmonization

Transcriptomic profiles, somatic mutation data, and clinical annotations for CRC were retrieved from The Cancer Genome Atlas (TCGA), encompassing paired tumor and adjacent normal specimens. External validation cohorts were acquired from Gene Expression Omnibus (GEO) repositories. Single-cell RNA sequencing data originated from the Tumor Immune Single-cell Hub (TISCH), while spatial transcriptomics derived from CRC_WholeTranscriptomeAnalysis_10x. Proteomic datasets were sourced from The Cancer Proteome Atlas (TCPA), with DNA methylation data accessed via the SMART application. All datasets underwent rigorous normalization to ensure cross-cohort comparability (cohort characteristics detailed in **Supplementary Table S1**).

### 2.3 Molecular subtyping and validation

Differential expression analysis of MRGs between CRC and normal tissues was conducted using transcriptomic datasets from TCGA, employing log2 fold change (|log2FC| > 1) with statistical significance (P < 0.05). Prognostic MRGs were identified by univariate Cox regression with two-sided P < 0.05; genes with positive (negative) coefficients were classified as risk (protective). Intersectional analysis yielded prognostic MLO-related differentially expressed genes (DEGs), which were subjected to functional annotation using Metascape for Gene Ontology (GO) terms and Kyoto Encyclopedia of Genes and Genomes (KEGG) pathways, with a significance threshold of P < 0.01 and a minimum enrichment count of 3. Protein-protein interaction networks were reconstructed using STRING (medium confidence score: 0.4) and visualized via string-db.org. Genomic alterations—including somatic mutations, copy number variations, and methylation patterns—were interrogated using the R package Maftools and GSCA database. Optimal patient clustering was determined by ConsensusClusterPlus R package, where the inflection point of the sum of squared errors (SSE) curve established cluster stability. Subtype validity was confirmed through principal component analysis (PCA) and Kaplan-Meier survival stratification.

### 2.4 Construction of an MLO-related prognostic signature

Lasso regression was utilized to identify robust prognostic MRGs. As a form of linear regression, Lasso regression incorporates a regularization term referred to as the λ penalty. This λ penalty induces sparsity in the model coefficients, causing many of them to be zero, which was a property that aids in pinpointing the most impactful features within high-dimensional datasets. Based on λ 1se, a six-MLO-gene MLO-related prognostic signature (MPRS) was developed in the training dataset. The MPRS was calculated as follows: MPRS = (-0.07488007 * CDC25C) + (-0.037942695*NRG1) + (0.014292142 *DMPK) + (0.031397958 *TIGD1)+(0.078577812 * TIMP1)+(0.192689886*ATP2A1) Here, “Expr” represents the expression levels of these six genes, and “Coef” denotes their corresponding coefficients. Subsequently, each individual in both the training and validation datasets was assigned an MLO index. The Predictor Of Naturally Disordered Regions (PONDR) algorithm was employed to assess the localization and functional roles of these MLO-associated proteins in LLPS. Patients in each dataset were divided into high-risk and low-risk groups, and principal component analysis (PCA) was performed to evaluate batch effects. Survival analysis was performed using Kaplan-Meier curves with log-rank testing (P < 0.05) to evaluate prognostic significance. The predictive accuracy of MPRS for overall survival (OS) was quantified via time-dependent ROC curve analysis, with the area under the curve (AUC) calculated at 1, 3, and 5 years to assess temporal performance.

### 2.5 Estimation of immune infiltration statuses

Immune infiltration profiles of CRC patients were evaluated using the ESTIMATE, CIBERSORT, and ssGSEA methods. The ESTIMATE algorithm, executed through the R package “estimate,” was used to calculate immune, stromal, and ESTIMATE scores for CRC patients (23). The CIBERSORT algorithm, with 1,000 permutations, was applied to determine the composition of 22 immune cell types. Additionally, the ssGSEA algorithm quantified the enrichment levels of 24 immune cell-related gene sets in CRC samples.

### 2.6 Single-cell sequencing technology and spatial transcriptomics

Single-cell RNA sequencing (scRNA-seq) data of CRC were retrieved from the TISCH database (**Supplementary Table S1**). Heatmaps illustrating gene expression profiles were constructed using the pheatmap R package. Unsupervised clustering of single cells was carried out using Seurat v4, which utilizes a graph-based clustering approach. We selected the top 2,000 variable genes using Seurat’s FindVariableFeatures (vst), performed PCA on the scaled matrix, and used the top 20 PCs for downstream neighbor graph construction and clustering. The FindClusters function was used on the top 10–25 principal components, and cluster identities were assigned using established cell-type marker genes. The UMAP algorithm was used to visualize high-dimensional single-cell data in two dimensions. Moreover, AUCell scores were calculated to quantify pathway activity heterogeneity across individual cells.

For spatial transcriptomics analysis, CRC_WholeTranscriptomeAnalysis_10x data in.h5 format and annotation results were downloaded from 10xGenomics. An enrichment score matrix was generated using the R package “Cottrazm,” and statistical significance was assessed using Wilcoxon Rank Sum Tests. Deconvolution analysis, combining spatial transcriptomics and single-cell data, was employed to accurately evaluate cellular composition at each location on 10x Visium slides. Using the get_enrichment_matrix and enrichment_analysis functions from the Cottrazm package, an enrichment scoring matrix was generated for further composition analysis. For visualization, Seurat’s SpatialFeaturePlot was used to display cell type enrichment scores. From the deconvolution results, the predominant cell type in each microregion was identified and visualized using Seurat’s SpatialDimPlot; SpatialFeaturePlot also illustrated the expression of specific genes across microregions.

### 2.7 Profiling of tumor immune ecotypes with Ecotyper

The EcoTyper machine learning platform (https://ecotyper.stanford.edu/) was employed to characterize cell type-specific states and multicellular communities. This computational framework leverages machine learning algorithms to enable large-scale identification of cell states and cellular ecosystems from bulk gene expression datasets. The relative abundance of each cell type was estimated based on the average abundance of its corresponding specific cell states (24).

### 2.8 The impact of MPRS on standalone ICI therapy groups

Data on copy number variations (CNVs), neoantigen load (NEO), and somatic non-silent mutations in TCGA were retrieved from UCSC Xena. The GISTIC 2.0 pipeline was used to analyze CNV characteristics. The R package “maftools” (version 2.12.0) was applied to visualize and compare tumor somatic mutation landscapes, as well as to identify genes with significantly different mutation frequencies between the two groups. Tumor Immune Dysfunction and Exclusion (TIDE) integrates expression profiles of T-cell dysfunction and exclusion in tumors with those of three cell types known to restrict T-cell infiltration: cancer-associated fibroblasts (CAFs), myeloid-derived suppressor cells (MDSCs), and M2 tumor-associated macrophages (TAMs) (25). The TIDE algorithm (accessed online at http://tide.dfci.harvard.edu/) was used to evaluate potential responses to immune checkpoint inhibitor (ICI) therapy, with input data as log2-transformed TPM-normalized RNA-seq counts. The area under the receiver operating characteristic curve (AUC) was calculated to assess the performance of target genes in predicting responses to immune checkpoint blockade (ICB), by comparing them with established immune response biomarkers such as tumor mutation burden (TMB), microsatellite instability (MSI), CD274, CD8, interferon gamma (INFγ), and TIDE. Three independent immunotherapy cohorts were included in this study: TCGA-COADREAD, GSE78220, and IMvigor210. Gene expression profiles were converted to TPM format using the `limma` R package to improve comparability, and the MPRS for each patient was computed to examine its correlation with ICI therapy response.

### 2.9 Drug sensitivity analysis

Drug sensitivity data were acquired using the R package “pRRophetic”. The 50% maximal inhibitory concentration (IC50) values were used to assess the sensitivity of cells to Gemcitabine and Etoposide. Additionally, the Genomics of Drug Sensitivity in Cancer (GDSC) and Cancer Therapeutics Response Portal (CTRP) databases were used to predict potential drugs associated with prognostic MLO-related differentially expressed genes (DEGs). For pharmacogenomic correlation with cetuximab. DepMap colorectal cancer cell lines (n = 28) were queried for cetuximab response from GDSC (AUC; higher AUC denotes lower sensitivity). NRG1 promoter methylation was computed as the methylation fraction averaged within 1 kb upstream of the TSS. Associations were evaluated by two-sided Spearman correlation. Parameters matched those used elsewhere in correlation analyses.

### 2.10 Prediction of intrinsically disordered regions and LLPS

Amino acid sequences of target proteins were retrieved from the UniProt database, and their disordered regions were analyzed using the PONDR platform. PhaSePred serves as a centralized resource for predicting both self-assembling and partner-dependent phase-separating proteins. It integrates scores from multiple phase separation-related prediction tools and provides proteome-level quantiles for various features. We employed this tool to profile phase separation propensity and extract valuable insights for identifying candidate proteins. FuzDrop, a sequence-based scoring method, predicts the likelihood of spontaneous LLPS in proteins, offering insights into protein functional relationships and deepening our understanding of such interactions. We utilized FuzDrop to assess protein behavior in condensed phases. The AlphaFold Protein Structure Database (AlphaFold DB), accessible at https://alphafold.ebi.ac.uk/, is a comprehensive public resource for accurate protein structure prediction (26, 27). To predict protein structures, we input the UniProt accession numbers of target proteins into this database, which was an AI system developed by DeepMind that predicts 3D protein structures from amino acid sequences. For proteins with multiple isoforms, canonical isoforms were selected as candidate sequences.

### 2.11 Quantification of NRG1-related pathway activities via proteomics and transcriptomics

Pathway enrichment analysis was performed on transcriptomic data from GEO and TCGA using the clusterProfiler R package, with mapping to known signaling pathways via KEGG and GO databases. Proteomic data were obtained from The Cancer Proteome Atlas (TCPA) projects. Correlation analysis was conducted to identify prognosis-related proteins associated with NRG1, using the criteria of p<0.05 and |Spearman’s R|>0.3. Reverse phase protein array (RPPA) data from the TCPA database were used to calculate pathway activity scores for 10 cancer-related pathways in CRC samples from TCGA (28). These pathways, included in the GSCA, are well-recognized for their association with cancer. The pathway activity score is defined as the sum of relative protein levels of all positive regulatory components minus those of negative regulatory components within a specific pathway. The GSVA score, which reflects the overall expression level of a gene set, exhibits a positive correlation with such expression.

### 2.12 Cell lines and tissue specimens

Human colorectal cancer cell lines (LoVo, SW480, RKO, HCT116) and the normal colon epithelial cell line NCM460 were obtained from the American Type Culture Collection (ATCC, Manassas, VA, USA). All cell lines were cultured in RPMI-1640 medium (Gibco BRL, Karlsruhe, Germany) supplemented with 10% fetal bovine serum, maintained at 37 °C in a 5% CO_2_ incubator. Immunohistochemical (IHC) staining was performed on human CRC tissues and their corresponding adjacent non-tumor tissues, which were collected from patients at Chongqing Beibei District Traditional Chinese Medicine Hospital. This study was approved by the Institutional Ethics Committee of Chongqing Beibei District Traditional Chinese Medicine Hospital.

### 2.13 qRT-PCR

Total RNA was extracted from cells using the TRIzol kit (Invitrogen). Quantitative real-time PCR (qRT-PCR) was carried out in accordance with standard protocols (29). The primers used for qRT-PCR are listed in **Supplementary Table S2**.

### 2.14 Immunohistochemistry

IHC staining was performed following established protocols on human CRC tissues and their adjacent non-tumor tissues, using an anti-NRG1 antibody (1:100, Immunoway, YT3054) (29). Detailed information of the patients involved is provided in **Supplementary Table S3**.

### 2.15 DNA methylation and rescue experiments

The MART (Shiny Methylation Analysis Resource Tool) App, available at http://www.bioinfo-zs.com/smartapp, is a web-based tool designed for analyzing DNA methylation patterns in human cancers (30). In this study, NRG1 methylation levels were analyzed using DNA methylation data via the SMART (Shiny Methylation Analysis Resource Tool) App (http://www.bioinfo-zs). The prognostic significance of NRG1 DNA methylation in CRC patients was explored using SurvivalMeth (http://bio-bigdata.hrbmu.edu.cn/survivalmeth/) (31). HCT116 and SW480 cells were treated with 10 μM Aza (Sigma-Aldrich, St. Louis, MO, USA) for 72 hours, after which total RNA was extracted for qRT-PCR analysis.

### 2.16 Statistical analysis

Spearman or Pearson correlation analyses were used to evaluate the relationships between continuous variables. For normally distributed variables, two-tailed t-tests or one-way ANOVA were applied to assess significant quantitative differences between or among groups, respectively. For non-normally distributed variables, the Wilcoxon test was used to compare differences between groups, while the Kruskal–Wallis test was employed for comparisons among multiple groups. To control for multiple testing, we applied the Benjamini–Hochberg method to control the false discovery rate (FDR) in all gene-level analyses, including differential expression, Cox regression, correlation, GSVA/ssGSEA modules, and ORA (reporting FDR q-values). For small families of ≤10 comparisons (e.g., limited clinical covariates), Bonferroni correction was used. For pathway-level RPPA panels, BH-FDR correction was applied. All statistical analyses were performed using R 4.1.2 and GraphPad Prism 9. Statistical significance was defined as FDR q < 0.05 (or Bonferroni-adjusted P < 0.05 for small families). Supplementary methods, tables, details of web tools, and R packages are available in the **Supplementary Material**. Specific information including versions, functions, access routes, parameters, and DOI numbers is provided in **Supplementary Table S4**.

## 3 Results

### 3.1 Differentially expressed and prognostic MRGs

**Figure 2:**
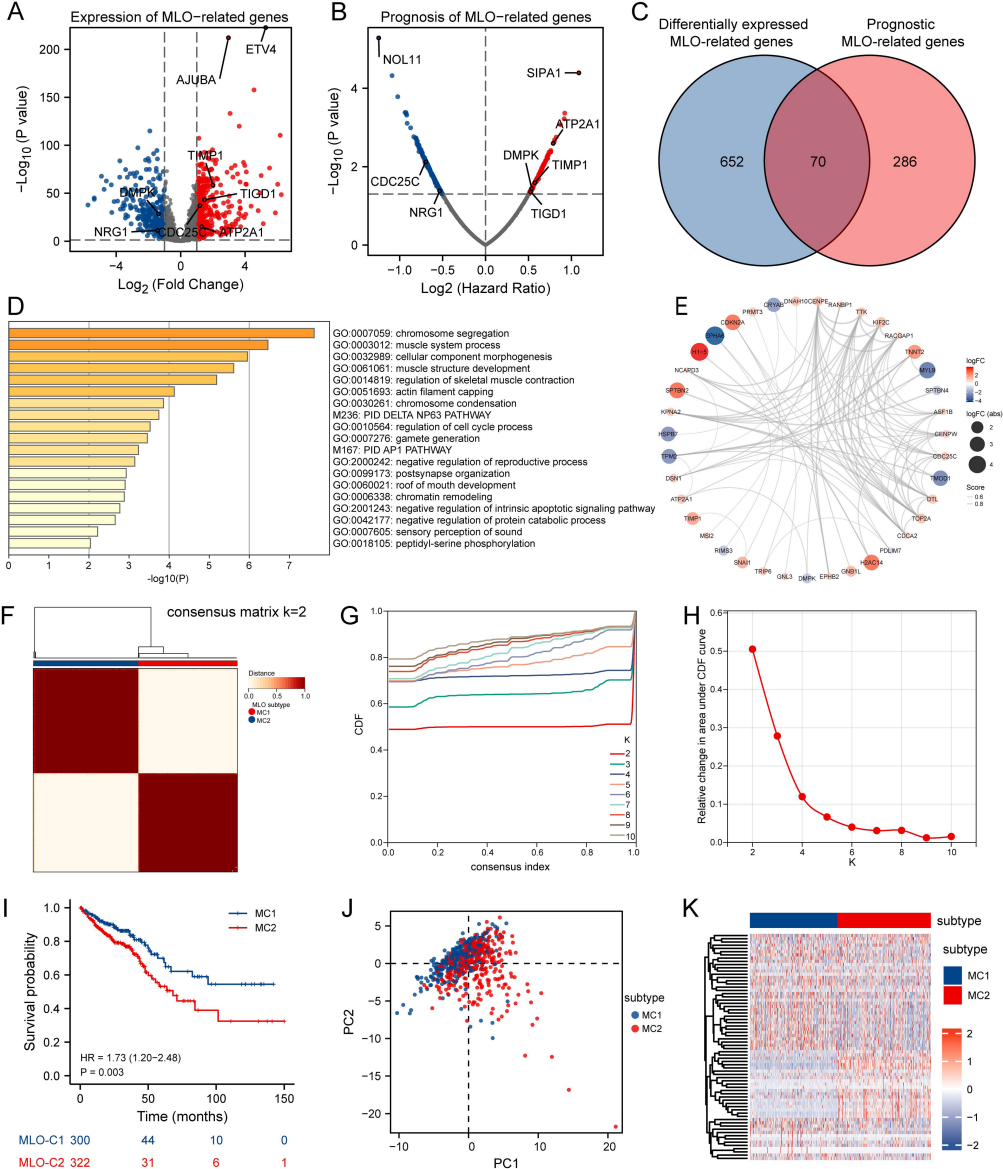
Identification of MLO-related Subtypes in CRC via Consensus Clustering. **(A)** A volcano plot displays differentially expressed genes (DEGs) with P < 0.05 and |log2FC| > 1.5 between colorectal cancer (CRC) tissues and adjacent normal tissues in the TCGA cohort, genes shown meet FDR q < 0.05. **(B)** A volcano plot of overall survival (OS)-related mRNAs in the TCGA dataset, where red indicates high-risk genes (Cox regression coefficient > 0) and blue indicates low-risk genes (coefficient < 0); genes shown meet FDR q < 0.05 after BH correction. **(C)** A Venn diagram identifies 70 prognostic MLO-related DEGs. **(D)** Gene Ontology (GO) and Kyoto Encyclopedia of Genes and Genomes (KEGG) enrichment analyses. **(E)** A protein-protein interaction (PPI) network of MLO-related DEGs. **(F)** A consensus map generated by non-negative matrix factorization (NMF) clustering. **(G)** A consensus cumulative distribution function (CDF) curve. **(H)** A CDF delta area curve, which illustrates the difference in area between the CDF under ki and the horizontal axis versus the CDF under ki + 1 and the horizontal axis. **(I)** Kaplan–Meier survival analysis showing significant differences in survival probabilities across MLO subtypes. **(J)** A t-distributed stochastic neighbor embedding (tSNE) plot visualizing the expression profiles of 70 prognostic MLO-related DEGs, effectively distinguishing between MLO subtypes. **(K)** A heatmap displaying the expression levels of 70 prognostic MLO-related DEGs across MLO subtypes.

Transcriptome data for 3737 MRGs were retrieved from the PhaSepDB database. A volcano plot revealed differential expression of 722 identified MLO-related DEGs, with 434 upregulated and 294 downregulated in CRC samples compared to adjacent normal tissues (**Figure 2A**). By intersecting these DEGs with prognostic MRGs identified via univariate Cox regression analysis, a total of 70 prognostic MLO-related DEGs were obtained (**Figures 2B, C**). Metascape enrichment analysis results, presented in **Figure 2D** highlights significant enrichment in terms including regulation of cell cycle process, PID AP1 pathway, negative regulation of intrinsic apoptotic signaling pathway, and negative regulation of protein catabolic process. A protein-protein interaction (PPI) network illustrated the complex regulatory relationships among these prognostic MLO-related DEGs (**Figure 2E**). **Supplementary Figure S1A** depicts the mutation profiles of prognostic MLO-related DEGs in CRC patients. Methylation levels of genes such as TRIP6, DSN1, POU2AF1, and EPHB2 showed a negative correlation with their expression (FDR < 0.05, **Supplementary Figure S1B**). Additionally, copy number variations (CNVs) of DSN1, CDCA2, GRINA, and ATP8B1 were positively correlated with their expression (FDR < 0.05, **Supplementary Figure S1C**). A total of 70 genes exhibited varying mutation frequencies in both READ and COAD samples (**Supplementary Figure S1D**). Missense mutations were the most prevalent, with single nucleotide polymorphisms (SNPs) occurring more frequently than insertions (INS) or deletions (DEL). The most frequently mutated genes were AHNAK2 and DNAH10, accounting for 28% and 24% of all CRC patients, respectively (**Supplementary Figure S1E**).

### 3.2 Identification of MLO related clusters in CRC

Consensus clustering analysis was performed using 70 prognostic MLO-related DEGs to investigate the association between MLOs and CRC subtypes. For clustering variables, k = 2 showed excellent stability, characterized by strong intragroup correlations and weak intergroup correlations, leading to the identification of two distinct clusters: MC1 and MC2 (**Figures 2F–H**). **Figure 2I** visually presents the distinct survival probabilities between the two clusters. PCA analysis confirmed that the two subgroups could be reliably distinguished (**Figure 2J**). A heatmap displayed the significantly different expression levels of the 70 prognostic MLO-related DEGs between the two groups (**Figure 2K**), while **Supplementary Figures S2A-D** clearly showed that the MC2 group had more mutations than the MC1 group. Additionally, notable differences in TMB, microsatellite instability, and neoantigen levels were observed between M1 and M2 (**Supplementary Figures S2E-G**). Taken together, these results strongly confirm the existence of two distinct MLO-related CRC subtypes.

### 3.3 Development and validation of the MLO-related prognostic model

**Figure 3:**
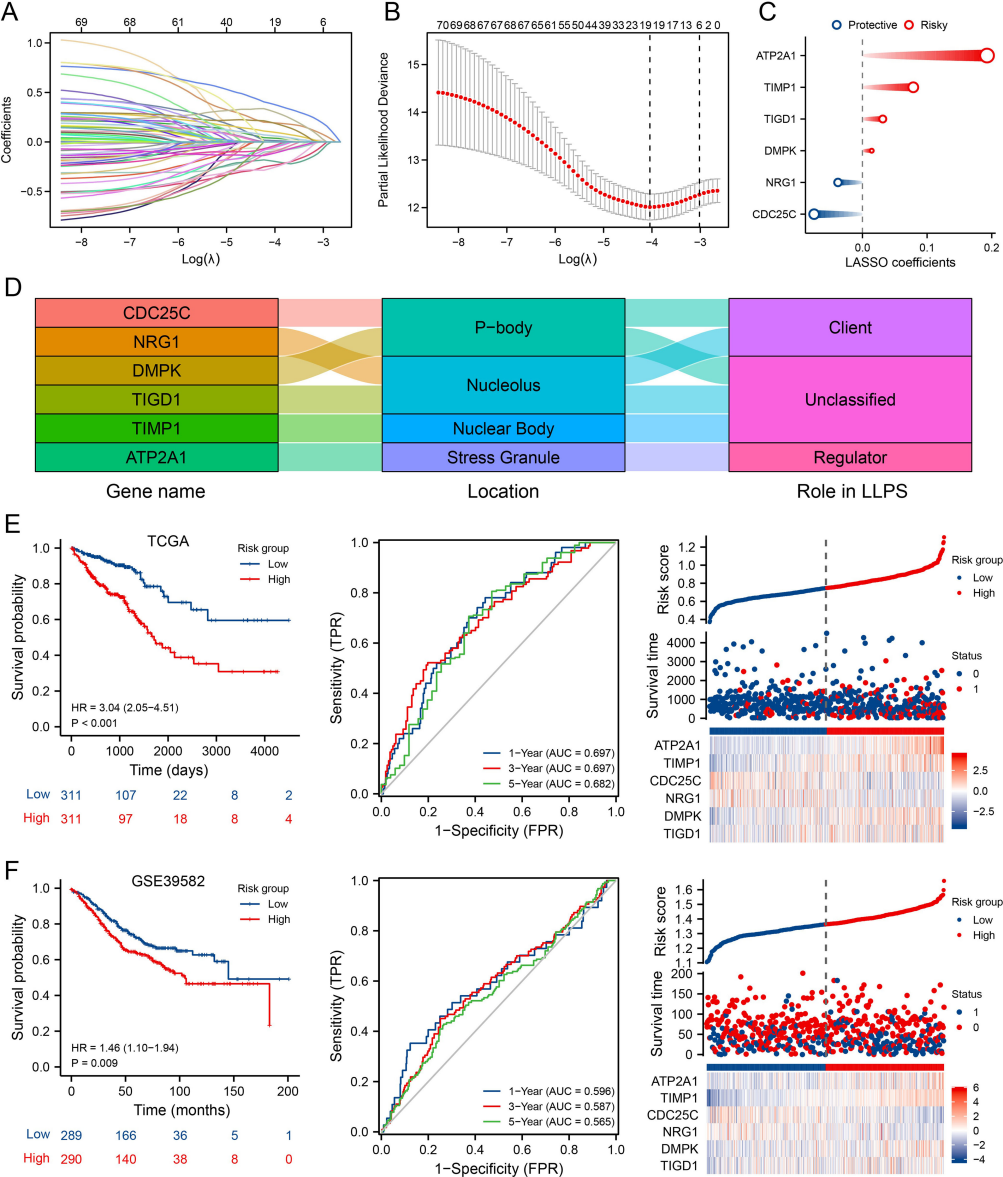
Development and Validation of the MLO-Associated Prognostic Model. **(A)** LASSO regression analysis of 70 prognostic MLO-related DEGs. **(B)** Cross-validation for selecting optimal genes, error bars = mean ± 95% CI across 10-fold cross-validation repeats. **(C)** LASSO coefficients for 6 selected MLO-related DEGs, circle size encodes absolute LASSO coefficient magnitude. **(D, E)** A mulberry diagram of 6 selected MLO-related DEGs/long non-coding RNAs (LRGs), accompanied by Kaplan–Meier survival analysis (OS), time-dependent ROC curves, and an overview of LLPS-related risk scores in the TCGA-COADREAD cohort. **(F)** Kaplan–Meier analysis for OS, time-dependent ROC curves, and summary plots of LLPS-related risk scores in the GSE39582 cohort.

To assist clinicians in predicting outcomes for CRC patients, we developed the MPRS prognostic model. To reduce overfitting, Lasso regression was applied to the prognostic MLO-related DEGs (**Figure 3A**). Using the optimal λ value and the lowest partial likelihood of deviance, the model was constructed with six genes and their corresponding correlation coefficients (**Figure 3B**). The formula is as follows: MPRS = (-0.07488007 * CDC25C) + (-0.037942695 * NRG1) + (0.014292142 * DMPK) + (0.031397958 * TIGD1) + (0.078577812 * TIMP1) + (0.192689886 * ATP2A1) (**Figure 3C**). PONDR analysis showed that the proteins encoded by these genes are distributed in p-bodies, nucleoli, nuclear bodies, and stress granules. Among them, CDC25C and DMPK function as clients, while ATP2A1 acts as a regulator (**Figure 3D**). **Figure 3E** presents a distribution plot highlighting significant differences in risk scores and model gene expression levels between the two risk groups. Identical analyses were conducted using the GSE39582 database (**Figure 3F**), where the high-risk group showed worse overall survival than the low-risk group. Kaplan-Meier survival analysis and PCA verified the significant differences between the groups. Additionally, validation was performed in six independent cohorts (TCGA_CRC, GSE17536, GSE103479, GSE87211, GSE39582, GSE28722), confirming the model’s robustness (**Supplementary Figure S3**). These results demonstrated that the prognostic signature is accurate, independent, and widely applicable.

### 3.4 Correlation of MPRS with cancer TIME patterns and ecotypes

**Figure 4:**
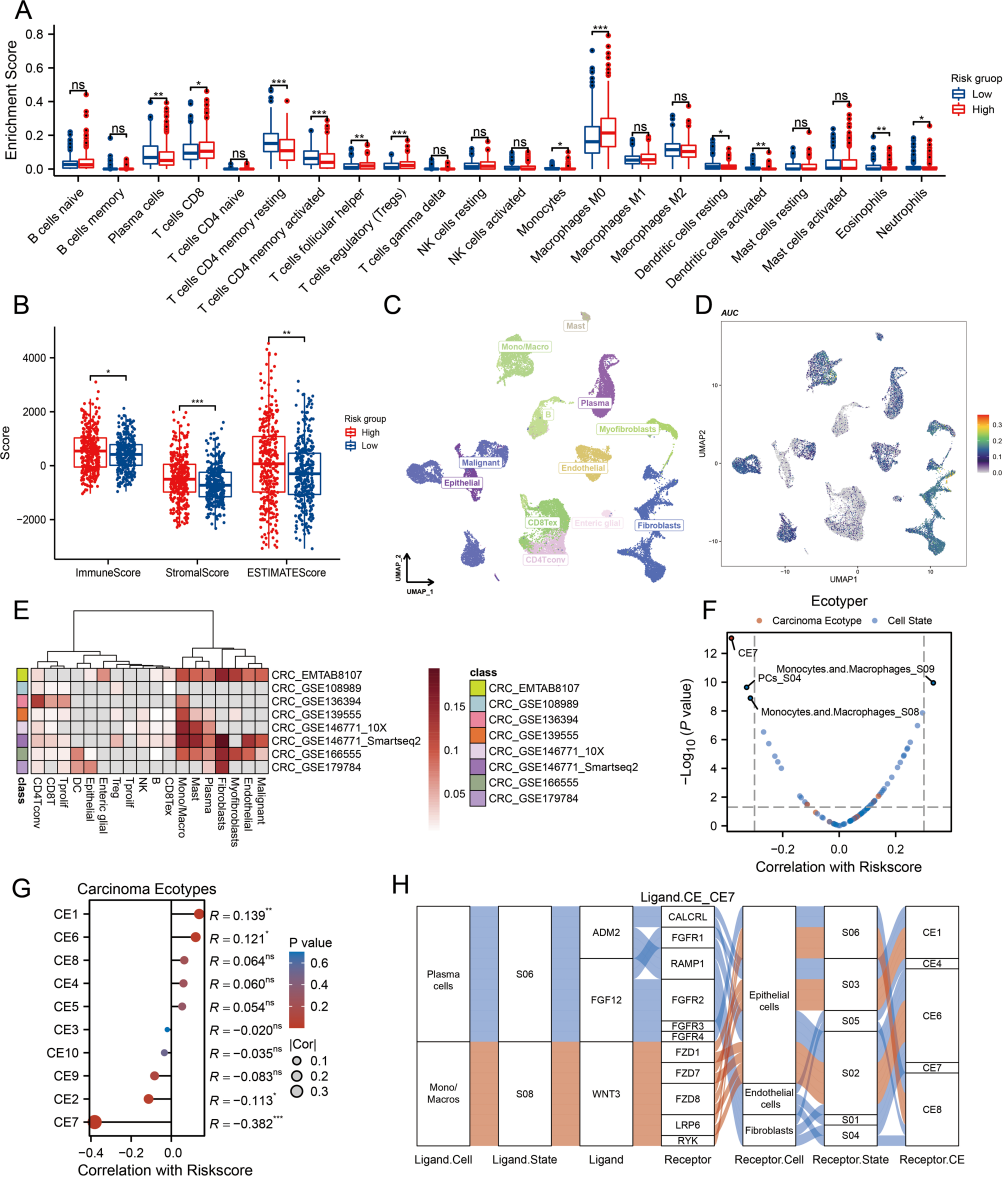
Association of MPRS with Immune Cell Infiltration, Single-Cell Profiles, and Ecotype Landscape in CRC. **(A)** Differences in the proportions of immune-infiltrating cells as determined by CIBERSORT. **(B)** ESTIMATE scores across subgroups. **(C)** A UMAP plot of single cells from CRC samples. **(D)** Expression of model genes in each cell type on the UMAP plot. **(E)** AUCell scores of different cell clusters. **(F, G)** Correlation between risk scores and cell ecotypes. **(H)** A Sankey diagram showing carcinoma ecotypes and cell states identified by the EcoTyper algorithm. Statistical two-group comparisons by Wilcoxon rank-sum (non-normal) or Student’s t (normality by Shapiro–Wilk) with Bonferroni adjustment where applicable, *p < 0.05, **p < 0.01, ***p < 0.001.

Given the growing recognition of MLOs’ potential role in regulating TIME patterns and influencing immunotherapy sensitivity (32, 33), we aimed to analyze TIME patterns across different MLO clusters. The distribution of enrichment scores from CIBERSORT and ssGSEA is presented in **Figure 4A** and **Supplementary Figure S4A**, revealing significant differences in both immune cell infiltration and immune functions among the MLO clusters. Notably, M0 macrophages showed greater infiltration in the high-risk group compared to the low-risk group. Using the ESTIMATE algorithm to assess TIME components in CRC patients, we found that the high-risk group exhibited the highest immune, stromal, and ESTIMATE scores relative to the low-risk group (**Figure 4B**). Traditional sequencing averages signals across a cell population, masking cell heterogeneity. Thus, we employed single-cell sequencing to capture heterogeneity unobtainable from bulk sequencing. UMAP results clearly showed distinct cell populations separated by their expression profiles (**Figures 4C, D**). **Figure 4E** illustrates that the six hub genes are primarily expressed in monocytes/macrophages, fibroblasts, mast cells, plasma cells, myofibroblasts, endothelial cells, and malignant cells. Additionally, we used the EcoTyper algorithm to identify and validate cell states and ecotypes. Monocytes/macrophages in state 09 showed a significant positive correlation with risk scores, while those in state 08 were negatively correlated (**Figure 4F**). Spatial transcriptomic analysis was performed on tissue specimens (**Supplementary Figure S6A**). After deconvolution, the cellular composition at each spot and spatial localization of all cell types are presented in **Supplementary Figures S6B, C**, revealing significant macrophage infiltration at the tumor-stroma junction. EcoTyper showed that risk scores were most closely associated with CE7 status (**Figure 4G**). Integrating known ligand-receptor interactions of CE7, potential cellular communication networks are shown in **Figure 4H**. Collectively, our comprehensive analysis demonstrates that MPRS is associated with distinct TIME patterns in CRC, with high-risk groups exhibiting enhanced immune cell infiltration and unique cellular ecotypes.

### 3.5 The significance of MPRS in forecasting the efficacy of ICI treatment

**Figure 5:**
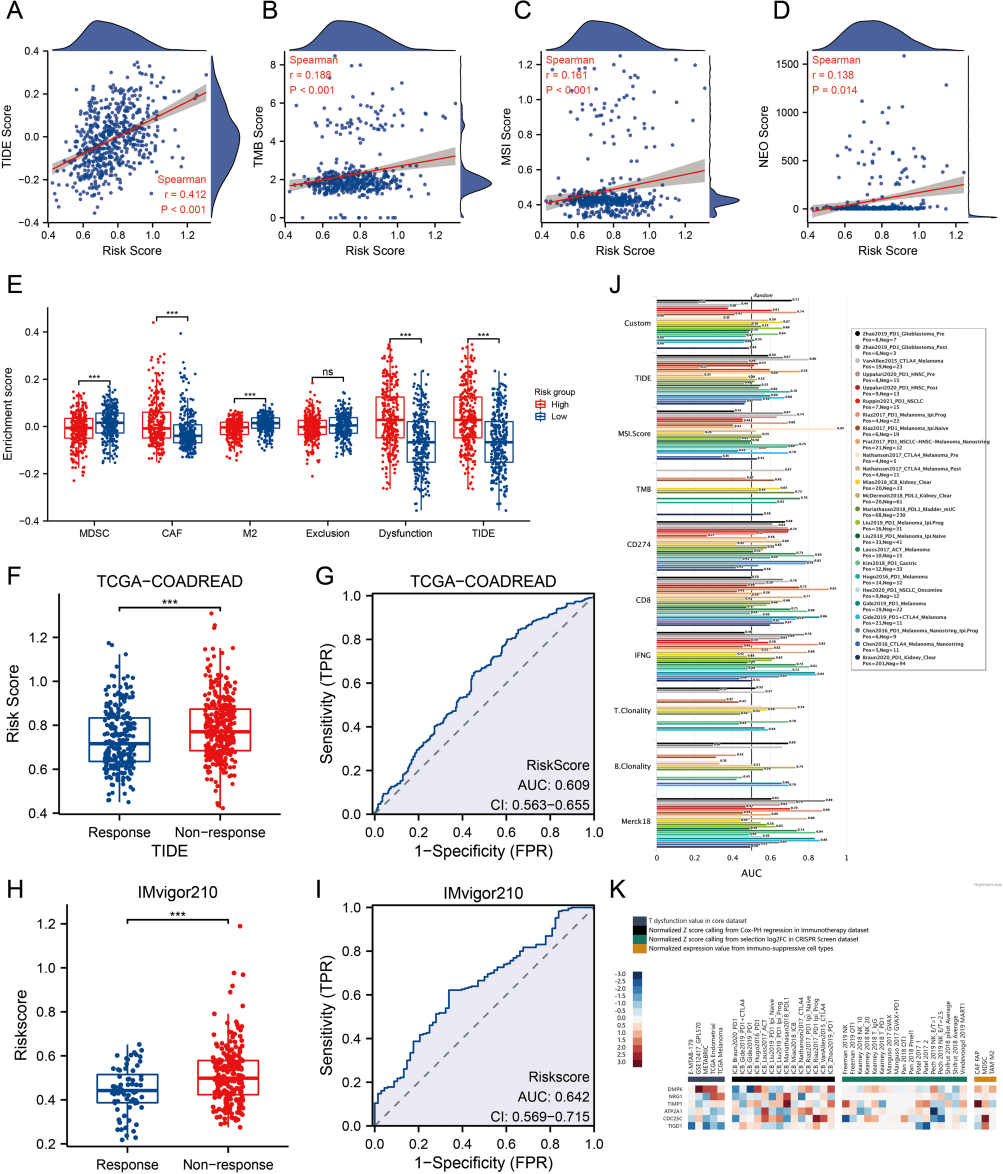
MPRS as a Novel Indicator for Immunotherapy Response. **(A)** Levels of TIDE scores across different MPRS groups. **(B)** Levels of TMB scores across different MPRS groups. **(C)** Levels of MSI scores across different MPRS groups. **(D)** Levels of neoantigen scores across different MPRS groups. **(E)** The enrichment scores of myeloid-derived suppressor cells (MDSC), cancer-associated fibroblasts (CAF), M2 tumor-associated macrophages (M2), T-cell exclusion, T-cell dysfunction, and TIDE between different MPRS groups. **(F, H)** Comparison of MPRS levels between subgroups with varying responses to anti-PD-L1 immunotherapy in the TCGA-COADREAD and IMvigor210 cohorts. **(G, I)** ROC curve illustrating the performance of the MPRS in predicting response to immunotherapy in the TCGA-COADREAD and IMvigor210 cohorts. **(J)** Bar plot displaying the AUC of MPRS and nine established immune response biomarkers across 25 immunotherapy cohorts. **(K)** A heatmap displaying T-cell dysfunction values, Z-scores, and expression levels of 6 prognostic MLO-related DEGs. Statistical two-group comparisons by Wilcoxon rank-sum (non-normal) or Student’s t (normality by Shapiro–Wilk) with Bonferroni adjustment where applicable, Spearman’s correlation with BH-FDR correction. *p < 0.05, **p < 0.01, ***p < 0.001.

We compared TIDE, TMB, MSI, and NEO scores across different risk groups, revealing that patients with high-risk scores had significantly higher levels of these scores than those with low-risk scores (**Figures 5A-D**, p<0.01). For TIDE, the high-risk group showed a higher dysfunction score, while no significant difference was observed in the exclusion score between the two groups, suggesting that CRC immune evasion may be associated with CD8+ T cell dysfunction (**Figure 5E**). To validate the utility of the risk score in predicting survival and immunotherapy response, we conducted separate analyses in two immunotherapy cohorts. Results from these cohorts indicated that patients with high-risk scores had poorer responses to immunotherapy compared to the low-risk group (**Figures 5F–I**). We also benchmarked MPRS against nine established biomarkers: across 25 immunotherapy cohorts, MPRS achieved an AUC > 0.5 in 11 cohorts, with the highest predictive performance in the Zhao2019-PD1-Glioblastoma-pre cohort (AUC = 0.71) (**Figure 5J**). Additionally, the heatmap suggested that NRG1 and DMPK may contribute to CD8+ T cell dysfunction (**Figure 5K**). These findings underscore the value of MPRS in predicting immunotherapy outcomes for CRC patients.

### 3.6 Evaluation of the chemotherapy response and candidate compounds for sensitization by MLO related risk score

**Figure 6:**
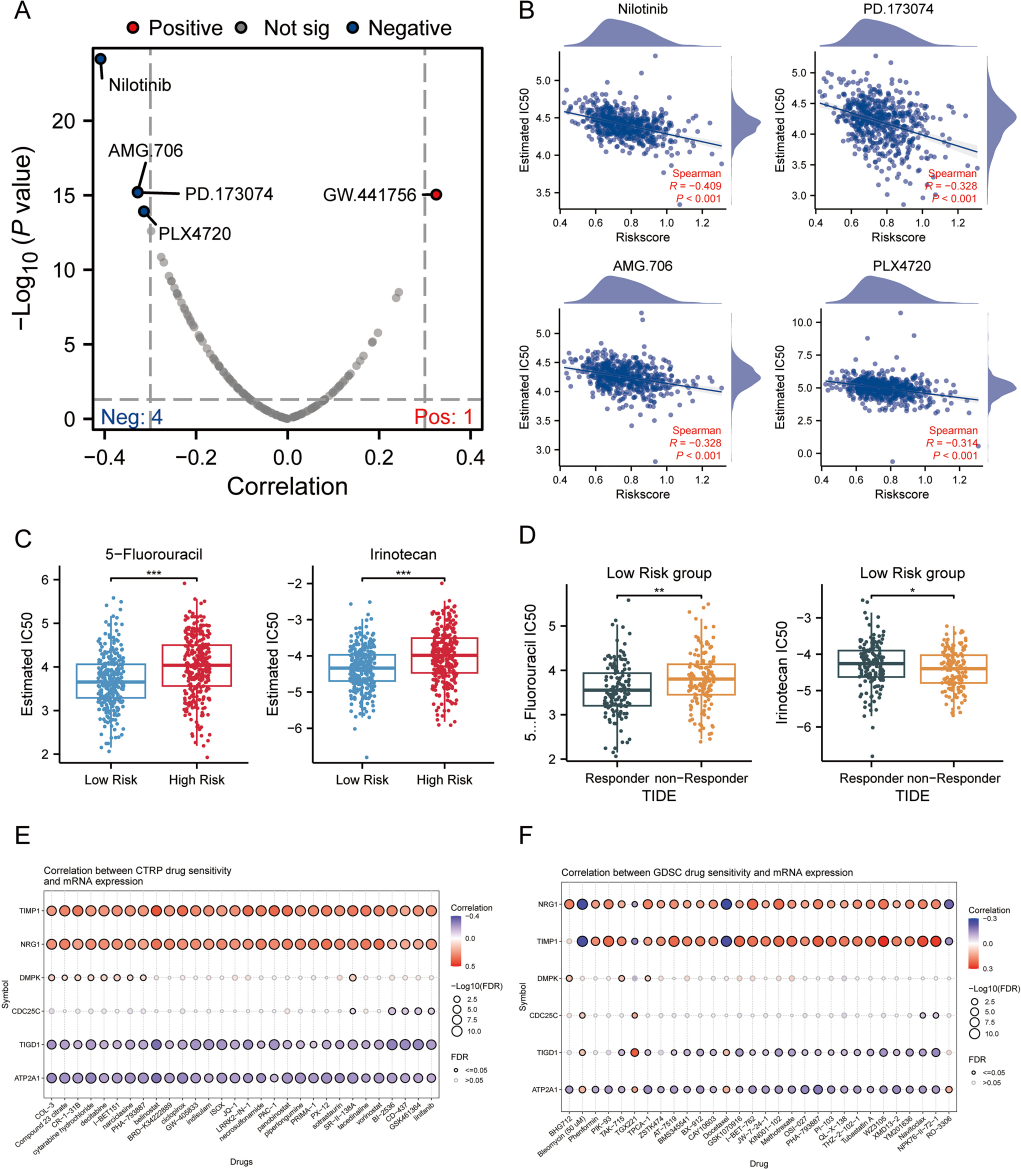
Association of MPRS with Chemotherapy Sensitivity and Candidate Compounds in CRC. **(A)** A volcano plot showing the sensitivity of CRC to chemotherapeutic drugs. **(B)** IC50 values of four chemotherapeutic drugs: PD.173074, Nilotinib, AMG.706, and PLX4720. **(C)** IC50 values of 5-fluorouracil and Irinotecan predicted by the pRRophetic algorithm in high- and low-MPRS groups. **(D)** Predicted IC50 values of 5-fluorouracil and Irinotecan in the low-MPRS group via the TIDE algorithm. **(E, F)** Drug sensitivity of selected genes based on CTRP analyses. **(F)** Drug sensitivity of selected genes based on GDSC analyses. Statistical two-group comparisons by Wilcoxon rank-sum (non-normal) or Student’s t (normality by Shapiro–Wilk) with Bonferroni adjustment where applicable, Spearman’s correlation with BH-FDR correction. *p < 0.05, **p < 0.01, ***p < 0.001.

A volcano plot illustrating the sensitivity of CRC to chemotherapeutic drugs is presented in **Figure 6A**. The IC50 values of PD.173074, Nilotinib, AMG.706, and PLX4720 showed a negative correlation with MPRS, indicating their potential efficacy in patients with high MPRS (**Figure 6B**). 5-fluorouracil (5-Fu) and Irinotecan were selected to assess drug sensitivity in CRC patients across different risk scores. Results revealed higher IC50 values for both drugs in the high-risk group, suggesting that these two classical anti-tumor agents are more effective in patients with low MPRS (**Figure 6C**). The TIDE algorithm was used to evaluate the risk stratification of MPRS for the combination of two chemotherapeutic drugs with immunotherapy. We found that in patients with low MPRS, responders showed increased sensitivity to 5-fluorouracil but increased resistance to irinotecan. Thus, low MPRS indicated a potential beneficiary population for 5-fluorouracil combined with ICB therapy (**Figure 6D**). Furthermore, several potentially effective drugs for chemosensitization were identified through GDSC and CTRP analyses (**Figures 6E, F**). Collectively, these findings demonstrate that CRC patients with higher MPRS have reduced sensitivity to 5-fluorouracil and Irinotecan, whereas lower MPRS is associated with greater drug sensitivity, highlighting potential therapeutic compounds for CRC treatment.

### 3.7 Expression and phase separation probability of MRPS genes

**Figure 7:**
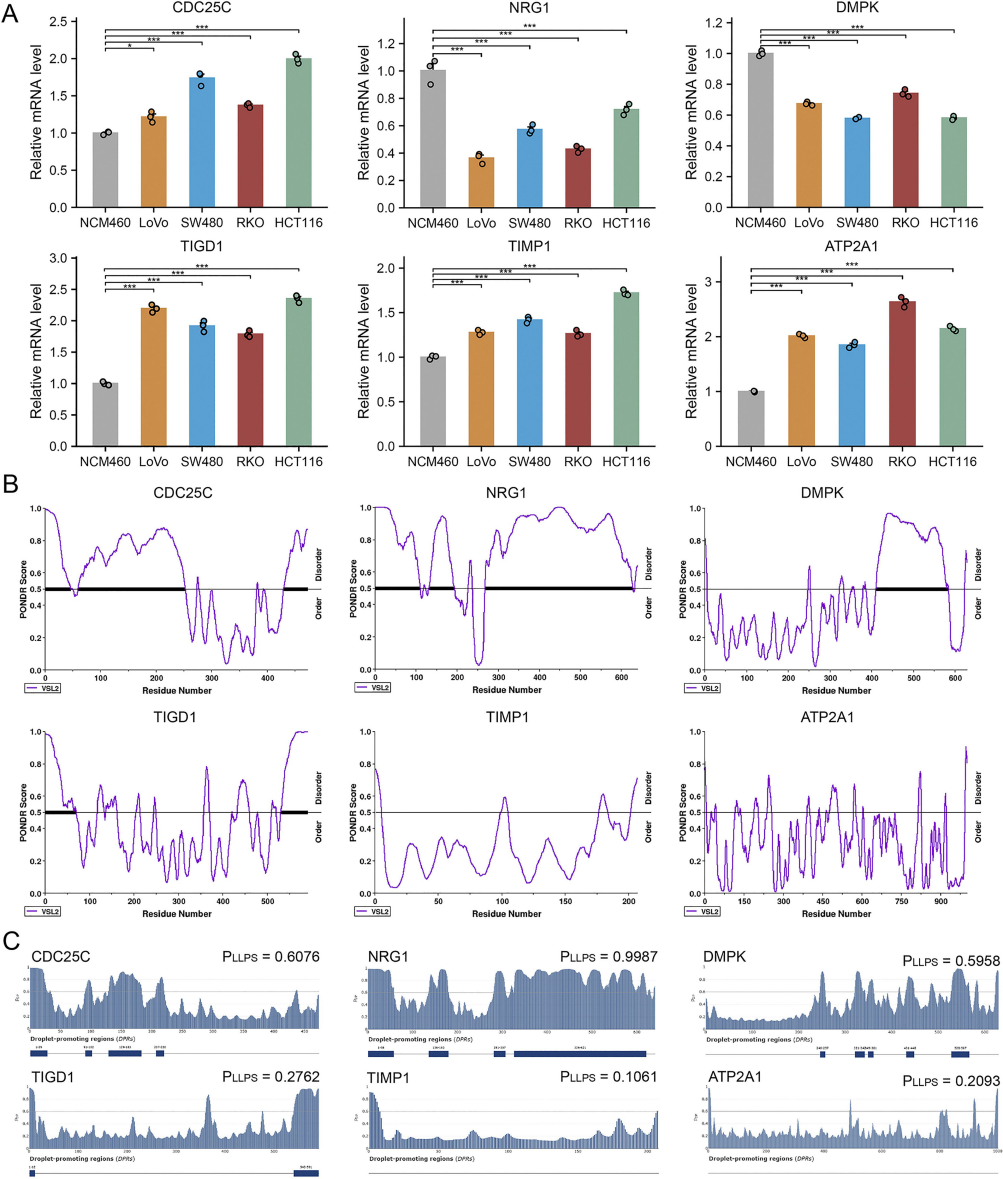
Expression in Cell Lines and LLPS Probability of MPRS Model Genes. **(A)** mRNA expression levels of model genes in normal and CRC cell lines assessed by quantitative PCR. **(B)** Identification of intrinsically disordered regions (IDRs) in model genes via PONDR. **(C)** Evaluation of phase separation potential of model genes via FuzDrop. Statistical comparisons performed using Wilcoxon or T tests with Bonferroni adjustment where applicable. *p < 0.05, **p < 0.01, ***p < 0.001.

**Figure 8:**
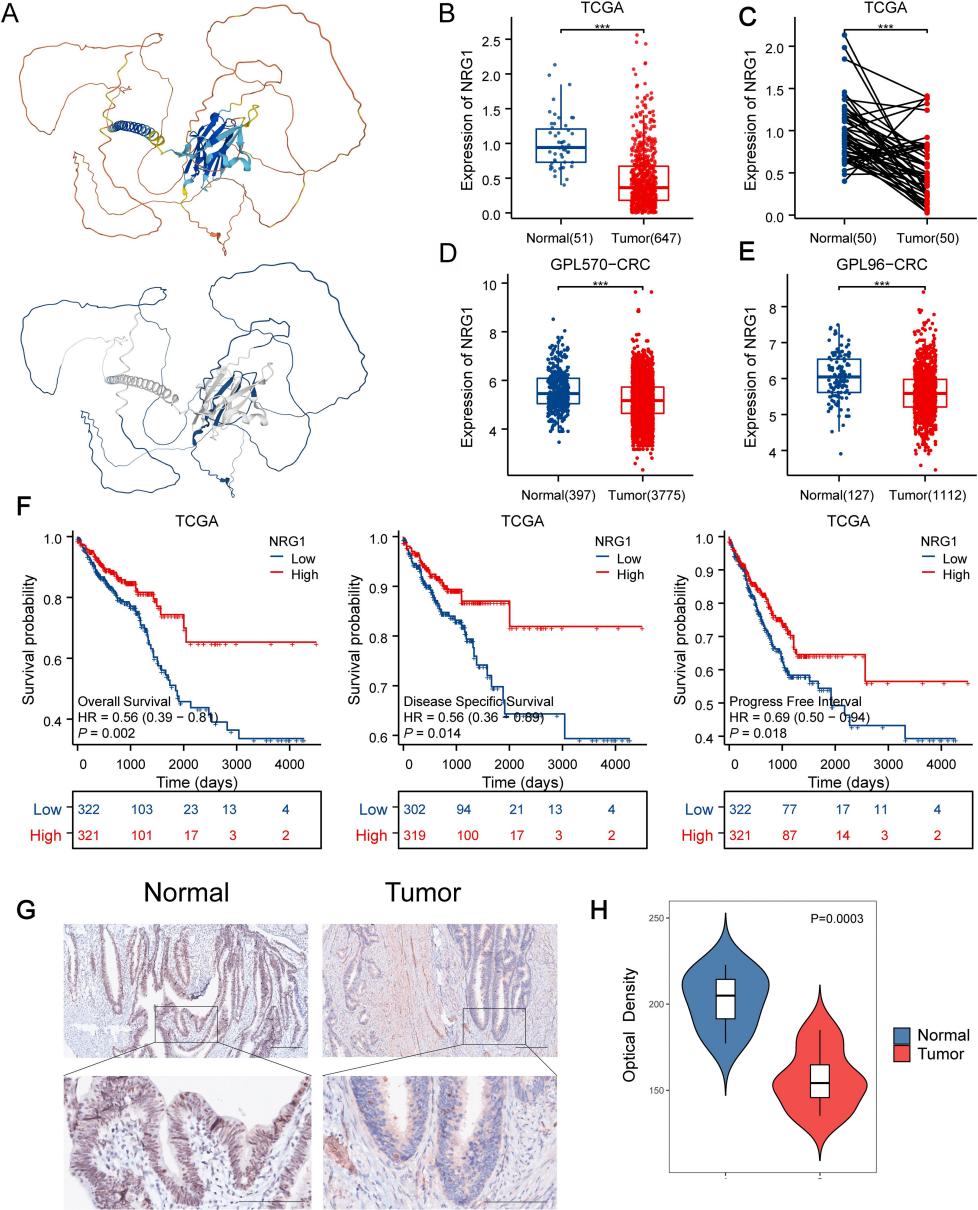
Downregulated NRG1 Expression in CRC Correlates with Poor Prognosis. **(A)** AlphaFold DB-predicted structural model of NRG1, colors indicate pLDDT (model confidence) and blue indicates the IDR region with low-pLDDT predicted by FuzDrop. **(B, C)** NRG1 expression levels in normal versus tumor tissues in TCGA datasets. **(D)** NRG1 expression levels in normal versus tumor tissues in GPL570 datasets. **(E)** NRG1 expression levels in normal versus tumor tissues in GPL96-CRC datasets. **(F)** Kaplan–Meier estimates of OS, DSS, and PFI for patients with different NRG1 expression levels in TCGA. **(G)** Immunohistochemical assay of NRG1 in CRC tissues and adjacent normal tissues (magnification ×100 and ×400). **(H)** Quantification of IHC images of CRC samples from 7 patients. Statistical two-group comparisons by Wilcoxon rank-sum (non-normal) or Student’s t (normality by Shapiro–Wilk) with Bonferroni adjustment where applicable. *p < 0.05, **p < 0.01, ***p < 0.001.

We validated the transcriptional levels of the six MPRS-related genes in both human colon cancer cell lines and normal colon epithelial cell lines. qRT-PCR analysis showed that the mRNA expression levels of TIMP1, CDC25C, ATP2A1, and TIGD1 were upregulated in cancer cell lines compared to normal cell lines, whereas NRG1 and DMPK exhibited reduced expression in cancer cell lines (**Figure 7A**). Using the PONDR tool, we detected high-scoring disordered regions in the proteins encoded by these six genes, suggesting their potential to undergo phase separation (**Figure 7B**). FuzDrop analysis further confirmed the phase separation propensity of these proteins, with NRG1 displaying the highest tendency (**Figure 7C**). The protein structure of NRG1, retrieved from the AlphaFold DB, revealed extensive IDRs, which further supports its strong potential for LLPS (**Figure 8A**). In the TCGA CRC cohort (n=514), KRAS mutation status showed no significant association with overall survival (P = 0.72) (**Supplementary Figure S5A**). However, KRAS mutant CRC patients exhibited distinct MPRS gene expression: NRG1 was downregulated (P<0.05) and DMPK upregulated (P<0.05) versus wild-type (**Supplementary Figure S5C**). KRAS mutant CRC patients also had higher MPRS scores (P<0.05) and elevated stress granule activity by ssGSEA (P<0.05) (**Supplementary Figure S5B**), indicating KRAS may driven MLO and SG dysregulation. Given that KRAS mutation modulates NRG1 expression while influencing MPRS and SG activity, NRG1 was further prioritized as the key MLO-related gene in CRC for further biomarker validation.

### 3.8 NRG1 protein influenced on progression and mediated the pathway activities via phase separation in CRC

**Figure 9:**
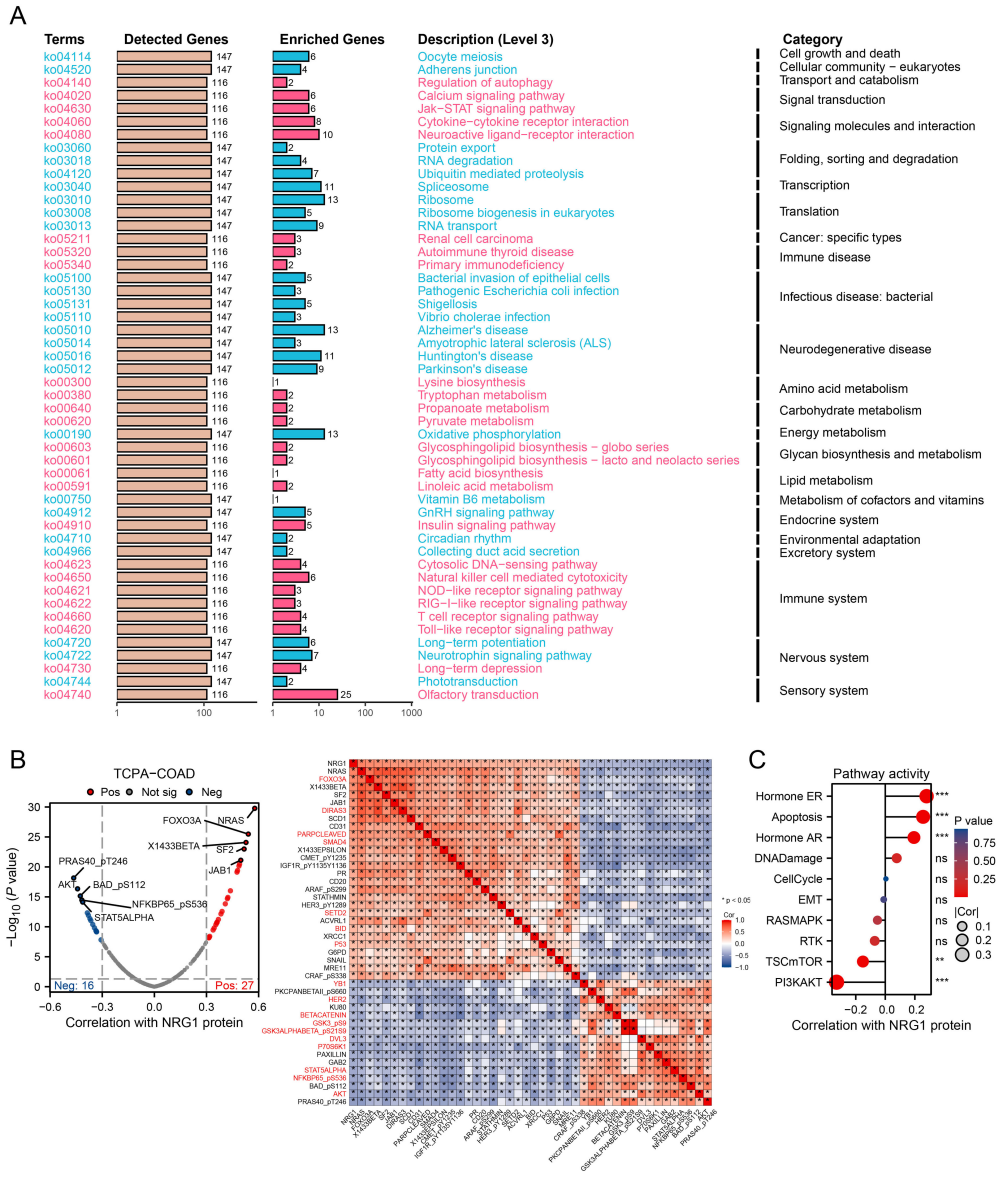
Integrating Transcriptomics and Proteomics to Unravel NRG1 Signaling. **(A)** A summary plot of over-representation analyses (ORA). **(B)** Proteins with expression significantly correlated with NRG1 protein levels in CRC (Spearman’s correlation coefficient > 0.3 or < -0.3, p < 0.05). **(C)** Pathway correlation analysis revealing relationships between pathway activity and NRG1 expression in the TCGA cohort. Spearman’s correlation with BH-FDR correction. *p < 0.05, **p < 0.01, ***p < 0.001.

Building on the identification of NRG1 as the most critical MPRS gene, we performed a comprehensive analysis of its clinical and pathological relevance in CRC. Our results revealed that NRG1 expression is significantly lower in tumor tissues compared to normal tissues in TCGA, GPL570_CRC and GPL96_CRC cohort (**Figures 8B-E**). Kaplan-Meier (K-M) survival curves showed that patients with high NRG1 expression had better disease-specific survival (DSS) and progression-free interval (PFI) in the TCGA cohort (**Figure 8F**), with similar trends observed in three additional independent cohorts (**Supplementary Figure S4B**). Moreover, we performed IHC assays on CRC tissue samples from 7 patients (containing tumor and adjacent normal tissue), we observed significantly down-regulated expression levels of the NRG1 protein in colorectal tumor tissues compared with adjacent benign tissues, consistent with our bioinformatic findings (**Figures 8G, H**; **Supplementary Figures S7A-F**). An over-representation analysis was conducted on genes associated with NRG1 (P<0.05), identifying 50 significantly enriched terms with FDR < 0.05 (**Figure 9A**). Key enriched terms included neuroactive ligand-receptor interaction, spliceosome, ribosome, Alzheimer’s disease, Huntington’s disease, oxidative phosphorylation, and olfactory transduction. Additionally, most genes were enriched in categories such as folding, sorting and degradation; infectious diseases (bacterial); neurodegenerative diseases; immune system; and nervous system (**Figure 9A**). Subsequently, univariate Cox regression analysis was used to evaluate the association between NRG1 and 223 proteins from the TCPA database. A volcano plot showed 27 proteins positively correlated and 16 proteins negatively correlated with NRG1. A heatmap further revealed that tumor-suppressive proteins (FOXO3A, DIRAS3, cleaved PARP, SMAD4, CD20, and P53) were positively correlated with NRG1, while oncogenic proteins (YB1, PKCβII, HER2, β-catenin, GSK3, and AKT) were negatively correlated with NRG1. This suggested that NRG1 exerts a protective role in CRC (**Figure 9B**). Pathway analysis indicated that NRG1 promotes apoptosis and inhibits oncogenic pathways such as mTOR and PI3K/AKT (**Figure 9C**). Collectively, these findings support that NRG1 functions as a tumor suppressor in CRC, where low expression is associated with poor prognosis and high expression correlates with improved survival.

### 3.9 Methylation of NRG1 in CRC

**Figure 10:**
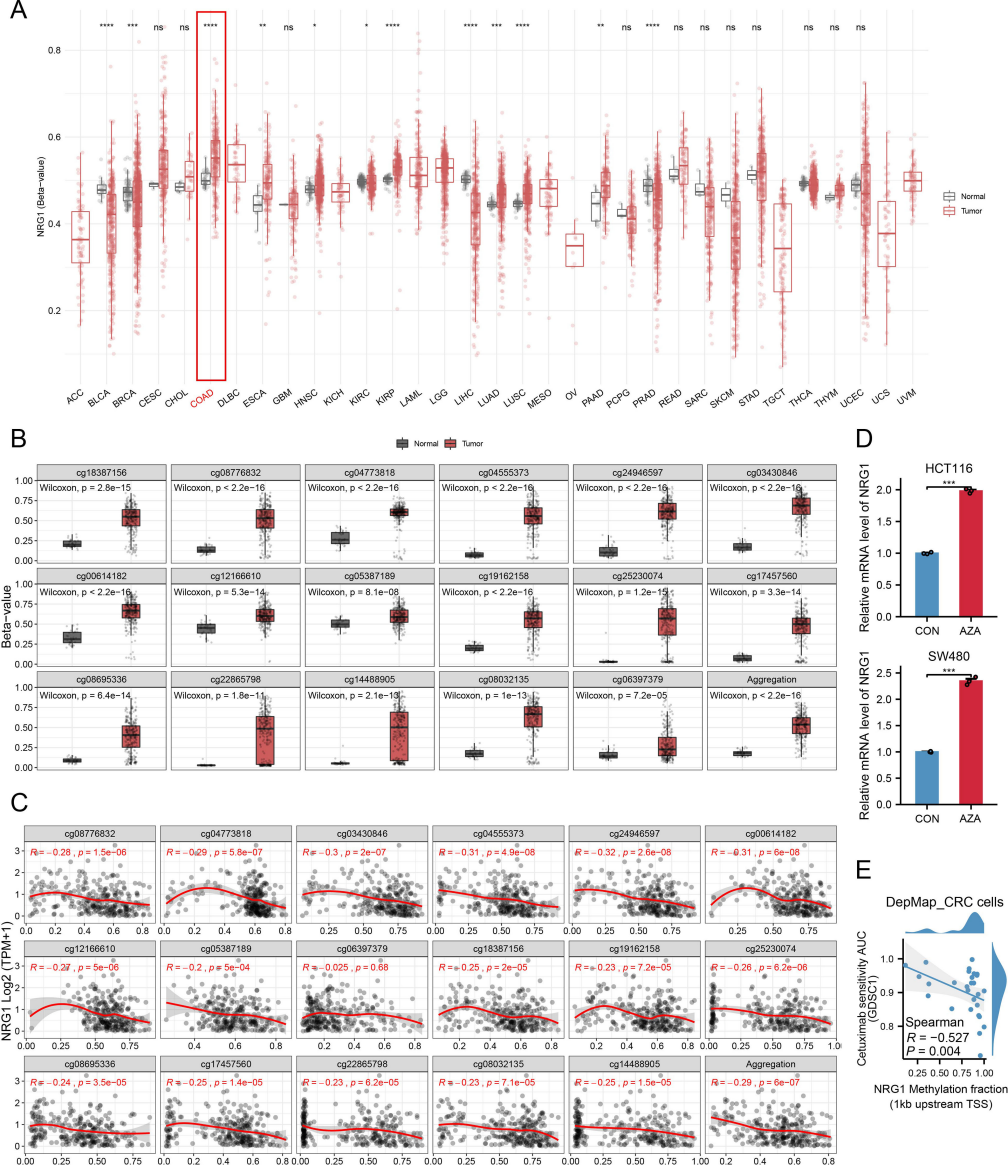
Promoter Methylation of NRG1 in CRC. **(A)** Methylation β-values of NRG1 across different tumor types and adjacent non-cancerous tissues in TCGA datasets. **(B)** Methylation levels of various probes in breast tumor and normal groups. **(C)** Analysis of the relationship between NRG1 expression and methylation sites (Spearman’s correlation coefficient, r). **(D)** NRG1 expression assessed by RT-PCR in HCT116 and SW480 cells after Aza treatment. Data are presented as mean ± SD from three independent experiments; significance levels: **(E)** Correlation between NRG1 promoter methylation (1 kb upstream of TSS) and cetuximab AUC (GDSC) in CRC cell lines (higher AUC = lower sensitivity). Statistical two-group comparisons by Wilcoxon rank-sum (non-normal) or Student’s t (normality by Shapiro–Wilk) with Bonferroni adjustment where applicable, Spearman’s correlation with BH-FDR correction. *p < 0.05, **p < 0.01, ***p < 0.001, ****p < 0.0001.

We explored the relationship between NRG1 methylation and its expression levels to clarify the mechanism underlying its reduced expression in CRC tissues. Using the SMART App, analysis via this platform showed that NRG1 methylation levels were significantly higher in CRC tissues than in adjacent normal colorectal tissues (**Figure 10A**). SurvivalMeth is a comprehensive platform for analyzing the prognostic significance of DNA methylation in cancer, which improves research efficiency by reducing redundancy. Using this tool, we investigated DNA methylation-related functional elements and found that 17 CpG sites in both the promoter and non-promoter regions of NRG1 had higher methylation β values in tumor samples compared to normal samples (**Figure 10B**). NRG1 mRNA expression was significantly negatively correlated with the methylation levels of the following probes: cg18387156, cg08776832, cg04773818, cg04555373, cg24946597, cg03430846, cg00614182, cg12166610, cg05387189, cg19162158, cg25230074, cg17457560, cg08695336, cg22865798, cg14488905, and cg08032135 (**Figure 10C**). Given the strong correlation between NRG1 promoter methylation and its expression, we further examined the effect of DNA methylation on NRG1 gene expression after bioinformatic analyses. Treatment with the DNA methyltransferase inhibitor Aza led to an upregulation of NRG1 expression (**Figure 10D**). Our findings suggest that changes in DNA methylation contribute to the downregulation of NRG1, and its expression increases following treatment with the DNA methyltransferase inhibitor Aza. These observations support NRG1 promoter methylation as a regulatory mechanism of its downregulation and nominate NRG1 demethylation as a candidate strategy that requires prospective validation and careful patient selection. To further evaluate the therapeutic implications, we analyzed DepMap CRC cell lines (n = 28) and found that NRG1 promoter methylation was inversely correlated with cetuximab AUC (Spearman *R* = - 0.527, *P* = 0.004) (**Figure 10E**), implying that NRG1 de-methylation/up-regulation may attenuate cetuximab activity via ERBB2–ERBB3 bypass under EGFR blockade.

## 4 Discussion

Mounting evidence suggests that membraneless organelles (MLOs) are pivotal to tumorigenesis and progression (34). Our hypothesis was that in-depth exploration of MLO-related biomarkers could significantly facilitate the identification of new tumor subtypes, as well as the prediction of prognosis and responses to immunotherapy. This study focused exclusively on colorectal cancer (CRC) patients. Through consensus clustering analysis of expression profiles from 70 prognostic MLO-related DEGs, we identified two distinct MLO subtypes in CRC patients. These subtypes exhibited significant differences in prognosis, genomic alterations, TIME patterns, and responses to immunotherapy. Using LASSO Cox regression, we constructed a prognostic signature (MPRS) to enable personalized comprehensive evaluation. Findings revealed that MPRS correlates with prognosis, genomic alterations, and TIME patterns in CRC patients, and demonstrated robust predictive capacity for responses to immune checkpoint inhibitor (ICI) therapy. Notably, we conducted detailed investigations into NRG1, one of the six core genes in the signature. Our results indicated that NRG1 has a marked propensity for phase separation and may influence CRC initiation and progression through methylation, thus establishing it as a novel methylation biomarker with predicted phase-separation propensity.

The development of distinct TIME patterns is a highly complex process involving multiple factors. Although progress has been made in certain areas, our overall understanding remains limited, requiring further research and innovative models to reveal its specific mechanisms and roles in cancer progression. Recent studies on MLOs have shed new light on their roles in TIME. Stress granules (SGs), a type of MLO formed under cellular stress, contribute to key cancer hallmarks such as proliferation, invasion, migration, apoptosis evasion, metabolic reprogramming, and immune evasion (35). Additionally, several MLOs in T cell transmembrane signaling receptors may cluster via phase separation to enhance signal transduction and regulate tumor immune responses (36). Given the critical role of MLOs in the cancer TIME, we reasoned that developing a prognostic signature to quantify TIME patterns in CRC patients could support personalized assessment. The constructed MPRS showed strong correlations with TIME patterns, key genomic alterations, prognosis, and ICI therapy responses in CRC patients. Specifically, the high MPRS group exhibited elevated immune, stromal, and ESTIMATE scores, indicating a higher abundance of non-tumor components, along with increased infiltration of tumor-infiltrating lymphocytes. Meanwhile, MPRS was significantly positively correlated with TMB, MSI, and NEO, further confirming higher immune infiltration in high-MPRS tumors. However, higher MPRS was associated with worse prognosis, which was explained by TIDE analysis: MPRS was significantly positively correlated with TIDE score. Although MPRS had no effect on CD8+ T cell exclusion, high-MPRS tumors showed significantly higher CD8+ T cell dysfunction. Therefore, high-MPRS phenotype was accompanied by a paradoxical immune landscape: despite “hot”, immune-infiltrated microenvironment features, it was characterized by CD8+ T cell dysfunction and abundant immunosuppressive cells such as regulatory T cells. What’s more, this immune-inflamed phenotype among high MPRS groups suggested a potential favorable response to immunotherapy, findings validated by subsequent predictions of ICI therapy responses. In conclusion, these results highlight the critical role of MLO-related subtypes in distinguishing TIME patterns and identifying patients likely to benefit from ICI therapy. Further research is warranted to clarify the precise mechanisms by which MLO-related processes shape specific TIME patterns.

Currently, research on membraneless organelles is still in its infancy, with a limited number of identified organelles and immature validation methods. Traditional hypothesis testing methods require screening one by one, consuming a large amount of manpower and time. In our study, we successfully identified six core MLO-related genes in CRC using multi-omics and AI technologies, with particular focus on the most promising Neuregulin 1 (NRG1). NRG1, a pivotal member of the epidermal growth factor (EGF) family, holds significant importance in cellular signaling pathways. Under normal physiological circumstances, the NRG1 protein undergoes proteolytic cleavage, resulting in the release of soluble NRG1 fragments that contain EGF-like domains. These soluble molecules participate in paracrine signaling by binding to ErbB3 or ErbB4 receptor subunits at a distance, which in turn induces the phosphorylation of their intrinsic kinase domains (37). Clinical investigations have shown that the NRG1 protein acts as an inhibitor in the progression of CRC (9–11). Nevertheless, the exact mechanisms behind its protective effects in CRC are not yet fully understood. Our research confirmed that low expression of NRG1 in CRC is significantly associated with an unfavorable patient prognosis. Additionally, we discovered that the NRG1 protein exhibits extensive IDRs and predicted phase-separation propensity, has a strong tendency to undergo phase separation, which may be a key mechanism influencing the progression of CRC. Further studies are needed to explore in detail how the LLPS processes involved in NRG1 assembly contribute to tumorigenesis and development.

In this study, we investigated the difference between KRAS-mutant and wild-type CRC patients. Prior studies have shown that oncogenic KRAS signaling enhances tumor cell fitness by promoting stress granule biogenesis (38). Our results are consistent with this model and further suggest a link to MLO dysregulation. Specifically, KRAS-mutant tumors exhibited decreased NRG1 expression and hyperactive SG formation, indicating that oncogenic mutations may intersect with subcellular organizational dynamics to sculpt tumor behavior. The translational relevance of NRG1 is strengthened as both a biomarker for risk stratification and a target for precision interventions in KRAS-mutant populations.

Abnormal DNA methylation plays a vital role in the initiation and progression of tumors. Our research suggests that methylation leads to the inhibition of NRG1 gene transcription in CRC. Azacitidine, a pioneering hypomethylating agent, is essential in the treatment of myelodysplastic syndromes and acute myeloid leukemia. Although targeting DNA methylation with hypomethylating agents has made significant progress in the treatment of various myeloid neoplasms (39, 40), its effectiveness in other solid tumors remains unclear. our data suggest that demethylation (e.g., with Aza) can upregulate NRG1 in CRC cell lines. However, our findings suggest a context-dependent effect of NRG1. Although reduced NRG1 expression is associated with poor prognosis at baseline, NRG1 upregulation under EGFR blockade may activate ERBB2–ERBB3 bypass signaling, thereby diminishing cetuximab efficacy. Therefore, NRG1 de-methylation should not be generalized as a therapeutic strategy for all patients. Instead, its application may be more suitable in non-anti-EGFR settings (e.g., RAS-mutant CRC, where EGFR antibodies are not used) or in combination with HER3/pan-ERBB inhibition. Furthermore, NRG1 methylation or expression could be developed as a biomarker to stratify patients for anti-EGFR therapy. Importantly, these conclusions are based on pharmacogenomic correlations in cell lines and require prospective validation in EGFR-dependent CRC models and clinical cohorts to stratify cetuximab use or rational combinations. Hence, NRG1 methylation/expression may serve as a biomarker to stratify anti-EGFR use and to nominate combinations with HER3/pan-ERBB inhibition.

This study presents several limitations that merit consideration. Firstly, the analyses were based on retrospective data from public databases; using prospective multi-center cohorts would lead to more reliable results. Secondly, although bioinformatics analyses provide valuable insights, experimental evidence is necessary to achieve a comprehensive understanding of molecular mechanisms, the anti-EGFR inference is pharmacogenomic and associative (DepMap) and requires perturbational validation in EGFR-dependent CRC models (NRG1 gain/loss ± HER3/pan-ERBB inhibition). Third, our LLPS-related analyses relied entirely on predictive algorithms without direct imaging or perturbation assays. Functional validation, including live-cell condensate imaging, FRAP assays, and stress-induced NRG1 perturbations, will be necessary to establish whether NRG1 indeed forms functional condensates in CRC.

In conclusion, our study proposed the MPRS prognostic signature for personalized comprehensive assessment. Compared with previous classifications of CRC patients, our MLO subtyping has advantages in revealing multi-dimensional heterogeneities, especially in terms of prognosis, genomic alterations, TIME patterns, and notably, responses to immunotherapy. Our study identified NRG1 as a methylation-linked biomarker with predicted phase-separation propensity in colorectal cancer. Future research can further verify and explore the phase separation and methylation characteristics of NRG1, providing new targets and therapeutic strategies for the targeted therapy of CRC.

## Data Availability

The original contributions presented in the study are included in the article/**Supplementary Material**. Further inquiries can be directed to the corresponding author.

## Glossary

Aza: 5-Aza-2-deoxycytidine
MLOs: membraneless organelles
LLPS: liquid–liquid phase separation
CRC: colorectal cancer
MPRS: MLO-related prognostic risk score
DEGs: differentially expressed genes
TCGA: The Cancer Genome Atlas
GEO: Gene Expression Omnibus
TISCH: Tumor Immune Single-cell Hub
TCPA: The Cancer Proteome Atlas
qRT-PCR: quantitative real-time PCR
IHC: immunohistochemistry
SMART: Shiny Methylation Analysis Resource Tool
OS: overall survival
DSS: disease-specific survival
PFI: progression-free interval
KEGG: Kyoto Encyclopedia of Genes and Genomes
GO: Gene Ontology
PPI: protein-protein interaction
LASSO: Least Absolute Shrinkage and Selection Operator
PONDR: Predictor Of Naturally Disordered Regions
FuzDrop: a sequence-based scoring method for predicting spontaneous liquid-liquid phase separation
AlphaFold DB: AlphaFold Protein Structure Database
CIBERSORT: Cell-type Identification By Estimating Relative Subsets Of RNA Transcripts
ssGSEA: single-sample gene set enrichment analysis
UMAP: Uniform Manifold Approximation and Projection
AUCell: Area Under the Curve for cellular enrichment
TIDE: Tumor Immune Dysfunction and Exclusion
TMB: tumor mutation burden
MSI: microsatellite instability
NEO: neoantigen
ICI: immune checkpoint inhibitor
ICB: immune checkpoint blockade
IC50: half maximal inhibitory concentration
GDSC: Genomics of Drug Sensitivity in Cancer
CTRP: Cancer Therapeutics Response Portal
IDRs: intrinsically disordered regions
SNPs: single nucleotide polymorphisms
INS: insertions
DEL: deletions
COAD: colon adenocarcinoma
READ: rectal adenocarcinoma
GSVA: Gene Set Variation Analysis
RPPA: reverse phase protein array
AUC: area under the curve
M0/M2: macrophage subsets (M0, M2)
MC1/MC2: MLO-related clusters 1/2
CNVs: copy number variations
HR: hazard ratio
FDR: false discovery rate
SSE: sum of squared errors
tSNE: t-distributed stochastic neighbor embedding
PCA: principal component analysis
GSCA: Gene Set Cancer Analysis
