# Supplementary material for: Membraneless organelles-based integrative analysis constructs an immune-related prognostic signature and identifies NRG1 as a novel methylation biomarker in colorectal cancer
Source: Front Immunol. 2025 Oct 20;16:1678096. doi: 10.3389/fimmu.2025.1678096 (PMC12580304; doi:10.3389/fimmu.2025.1678096)
Supplement: Supplementary file 1 [file DataSheet1.docx]

Supplementary Figures


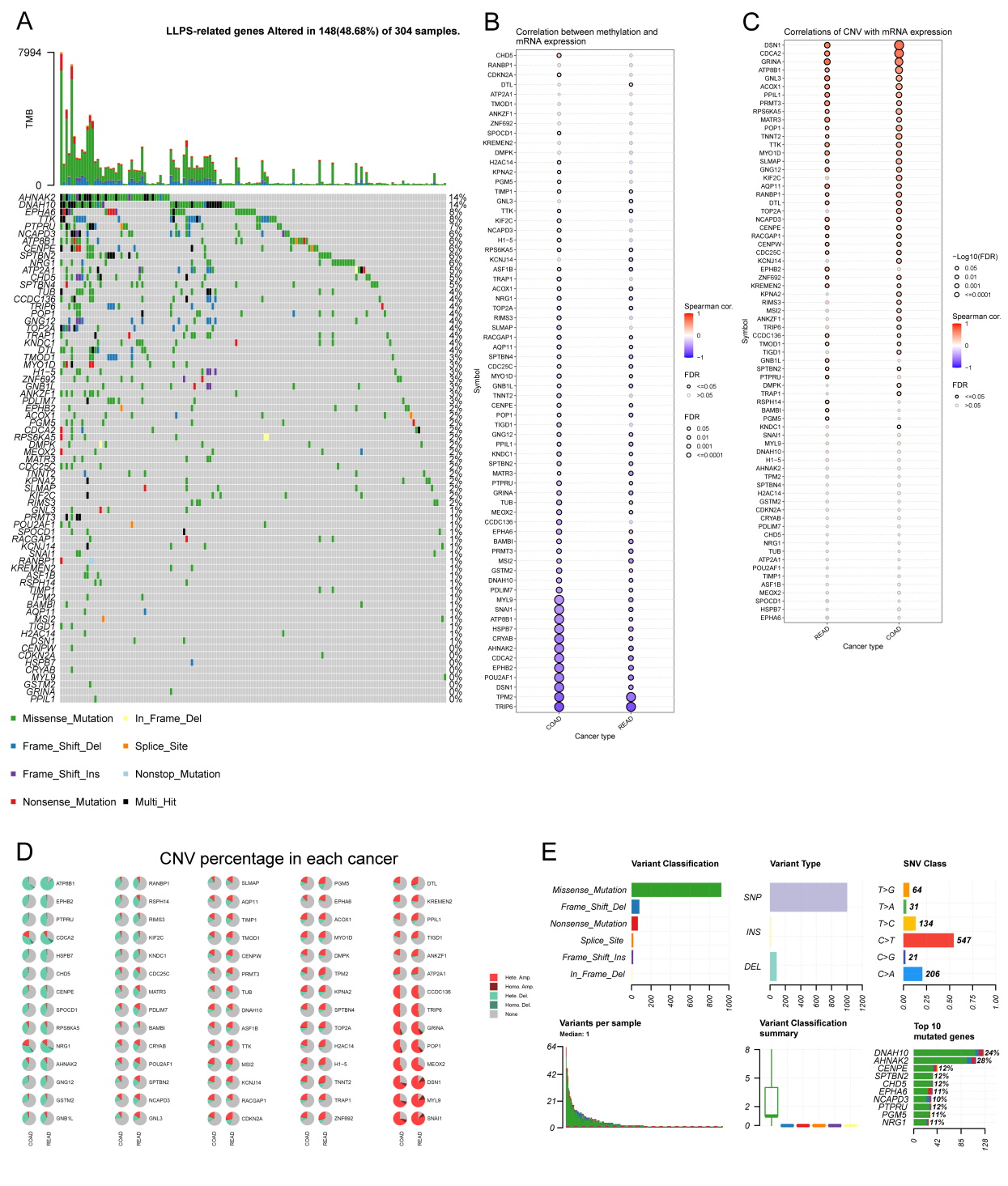


**Fig. S1 SNV and CNV landscapes of MLO-related prognostic genes.** (A) Mutation profiles of MLO-related genes;(B) The correlation between methylation and genes mRNA expression. (C) The correlation between CNV and genes mRNA expression. (D) Pie plot displays the global CNV profile of MLO-related genes in CRC. (E)The summary plot displays number of variants in each sample. SNV, single nucleotide variant; CNV, copy number variant; GSDMs, gasdermins.


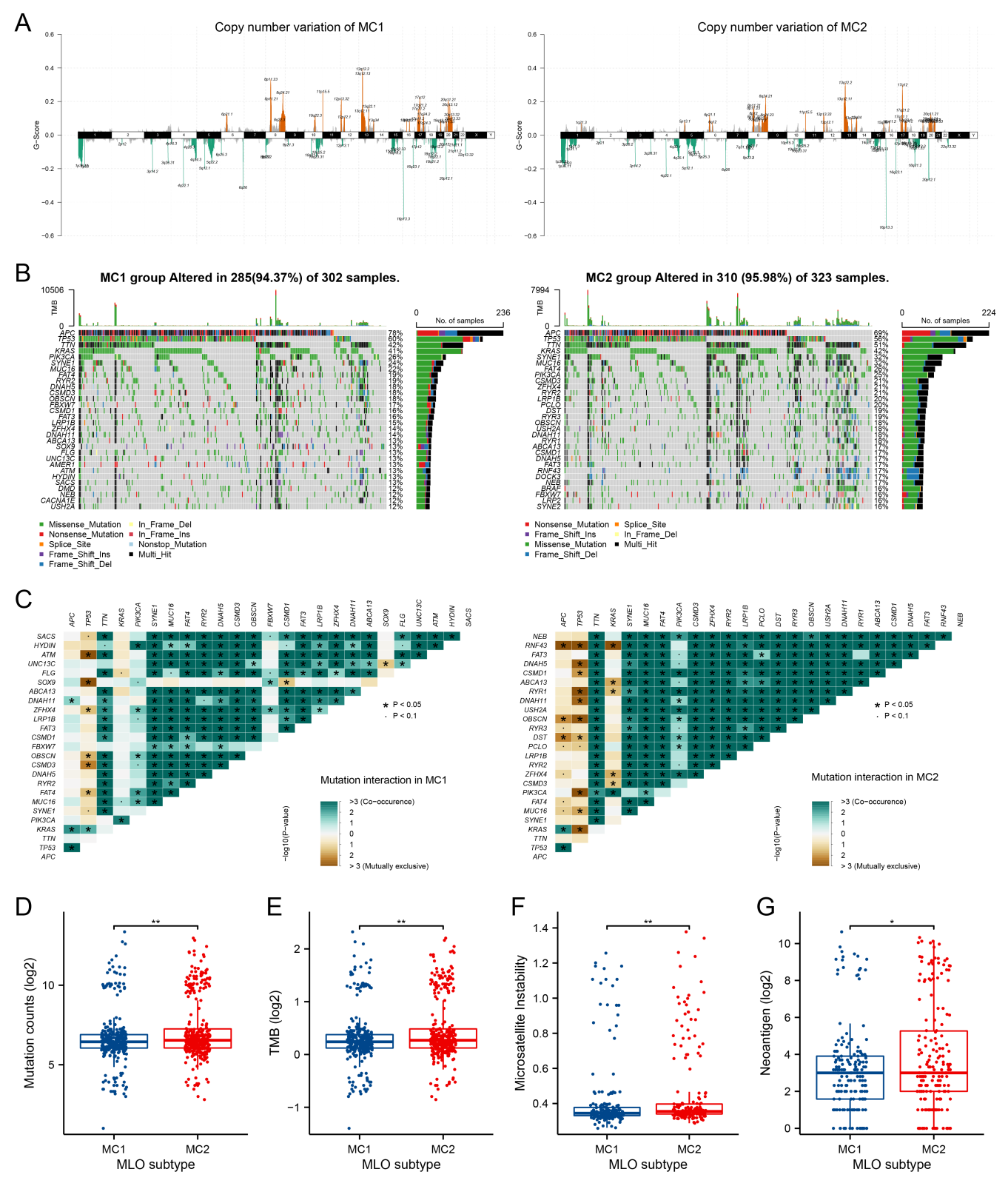


**Fig. S2: Variations of SNV and CNV in distinct MLO-related clusters.** (A) The copy number variation of MC1 and MC2;(B)the genetic alteration profiles in the MC1 and MC2 groups. (C)heatmaps of mutation interactions in MC1 and MC2. (D) Mutation counts among different MLO subtypes (E)TMB among different MLO subtypes (F)Microsatellite Instability among different MLO subtypes (G)Neoantigen among different MLO subtypes .


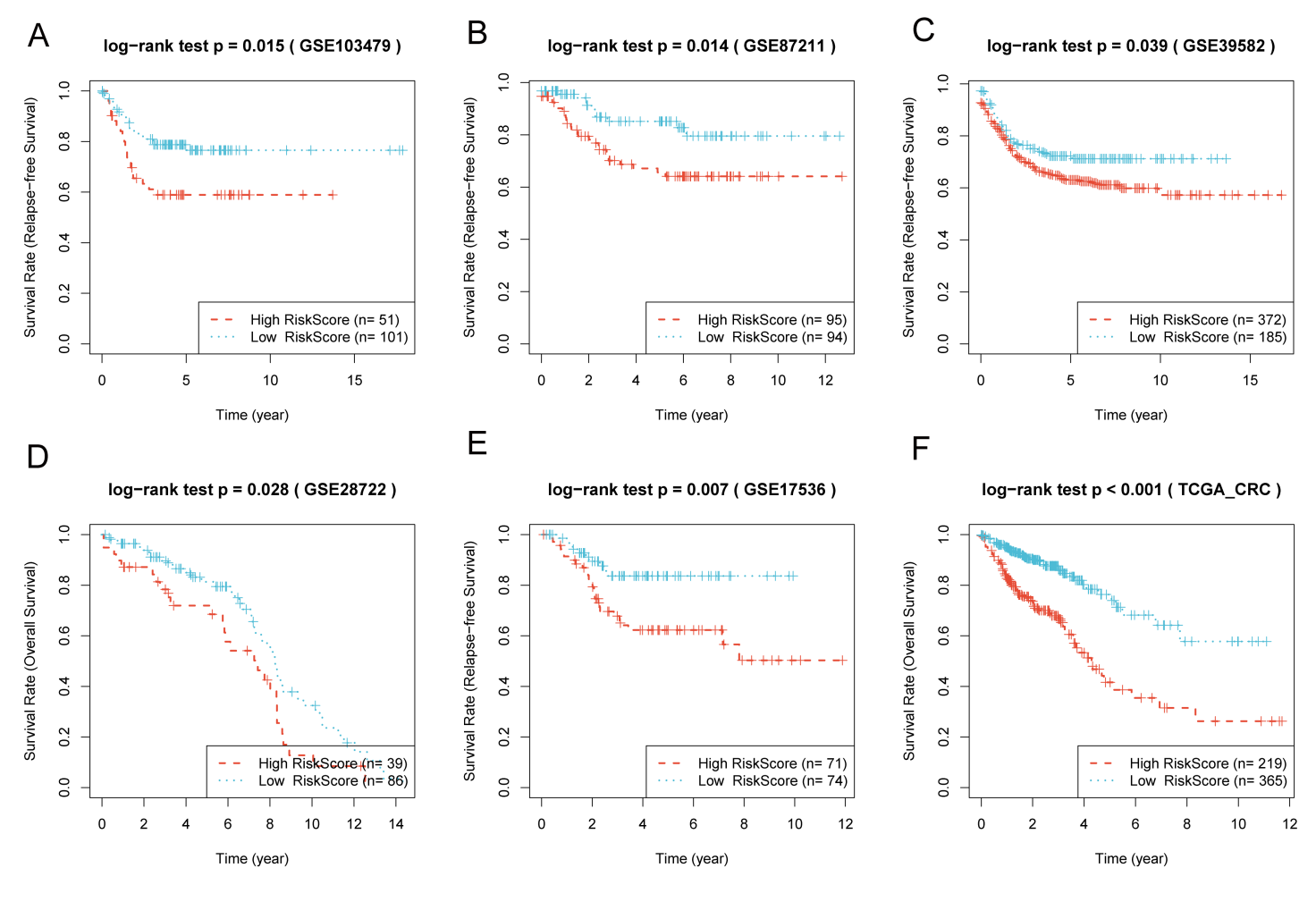


**Fig. S3: Survival analyses for validating the MPRS in various independent datasets.** (A-F) Kaplan - Meier survival curve comparing the survival outcomes between High RiskScore group and Low RiskScore group in GSE103479 (A), GSE87211 (B), GSE39582 (C), GSE28722 (D), GSE17536 (E) and TCGA (F).


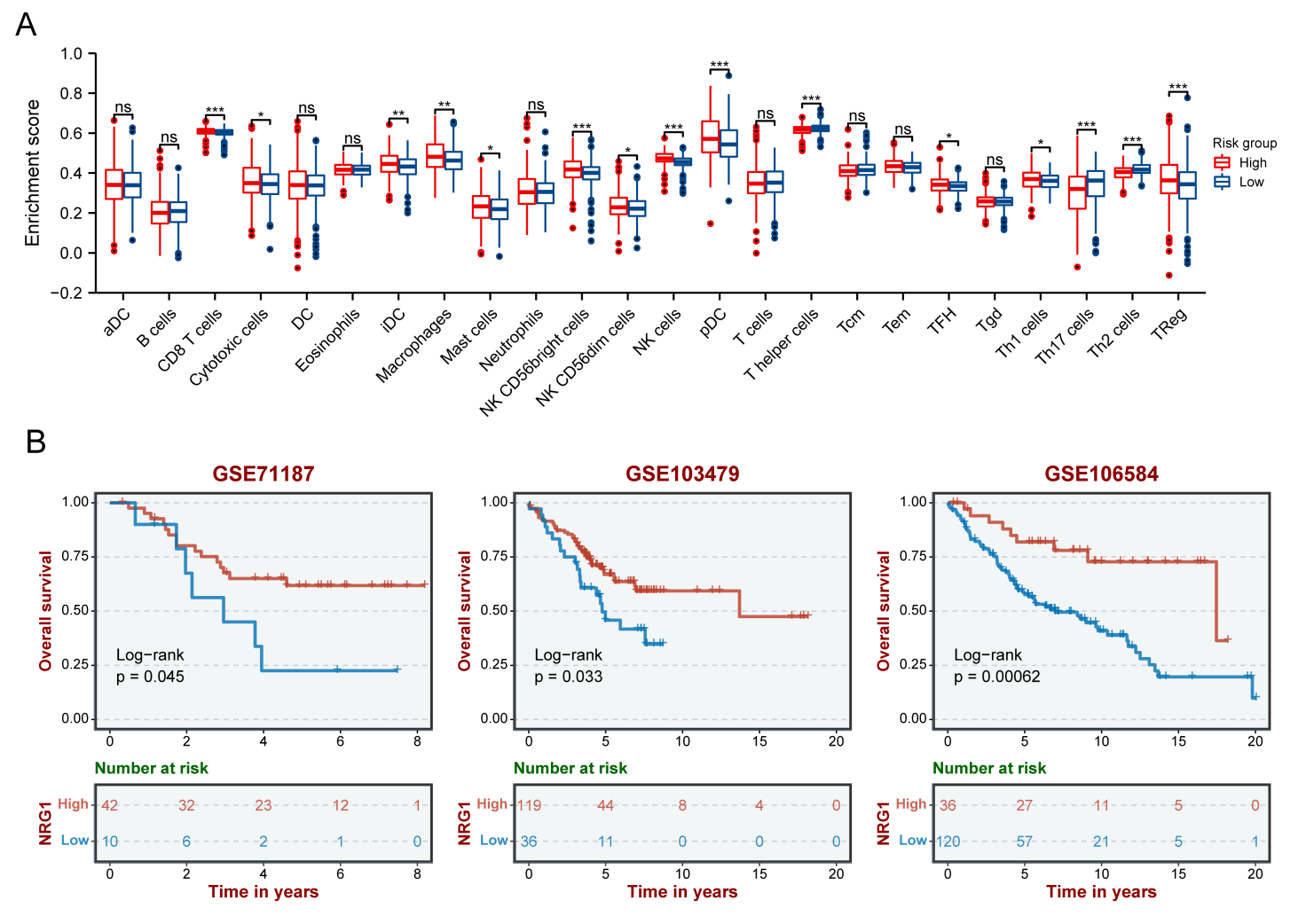


**Fig. S4: Immune infiltration and survival analysis of CRC patients.** (A)The enrichment scores for various immune cell types in high and low risk groups by ssGSEA.(B) Kaplan - Meier survival curves demonstrated the overall survival of patients in NRG1 high and low for three different datasets: GSE71187, GSE103479, and GSE106584.


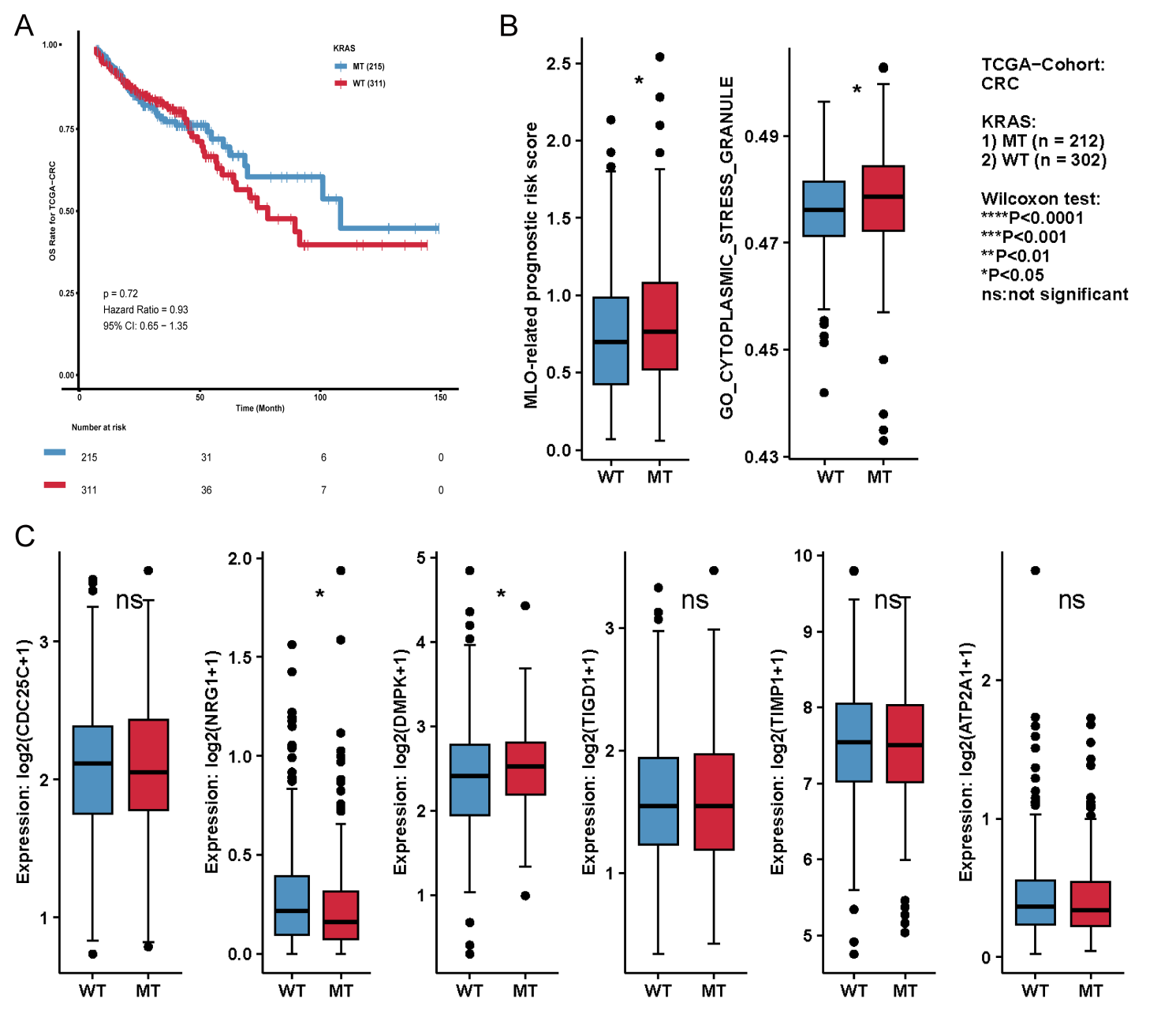


**Fig. S5: KRAS mutation-related survival, MPRS, and gene expression analysis in CRC patients.** (A) Kaplan-Meier survival curves demonstrated the overall survival of KRAS mutant (MT) and wild-type (WT) patients in the TCGA CRC cohort. (B) Left: MPRS-related prognostic risk scores in KRAS MT and WT patients; Right: ssGSEA enrichment scores for stress granule in KRAS MT and WT patients. (C) Box plots showing the expression of CDC25C, NRG1, DMPK, TIGD1, TIMP1, and ATP2A1 in KRAS MT and WT patients. Statistical two-group comparisons by Wilcoxon rank-sum (non-normal) with Bonferroni adjustment where applicable, *p < 0.05.


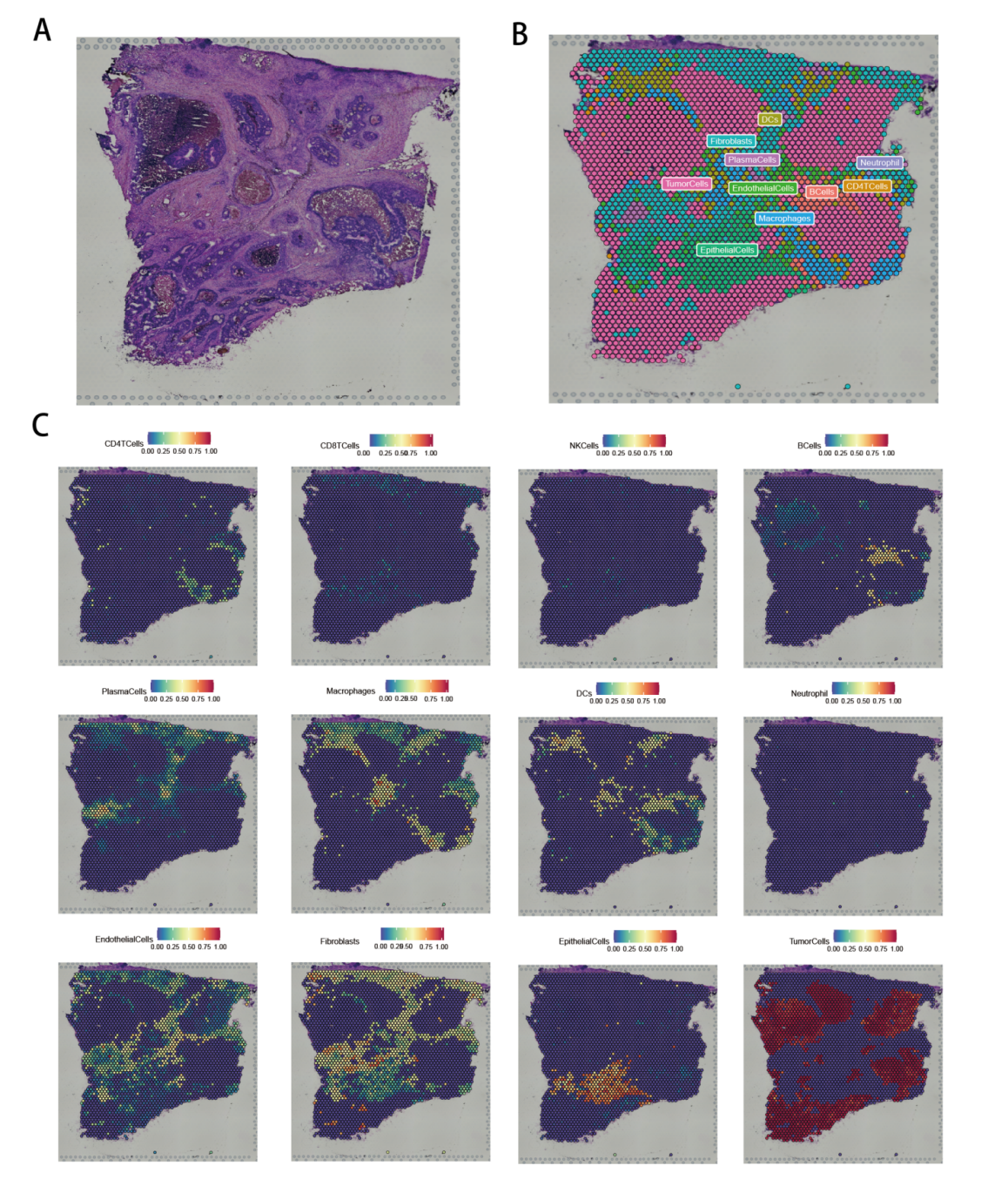


**Fig S6:Cell type inference of spatial voxels from spatial transcriptomics slides of CRC tissue.**

1. The corresponding histology CRC slide. (B) Each spotrepresents a microregion in spatial transcriptomics, with different colors signifying distinct cell types. (C) Gene expressions in the CRC tissue were profiled from 10-μm serial tissue sections by the 10X Genomics Visium platform. Color intensity is proportional to cell type-specific gene expression in the scRNA-seq dataset.


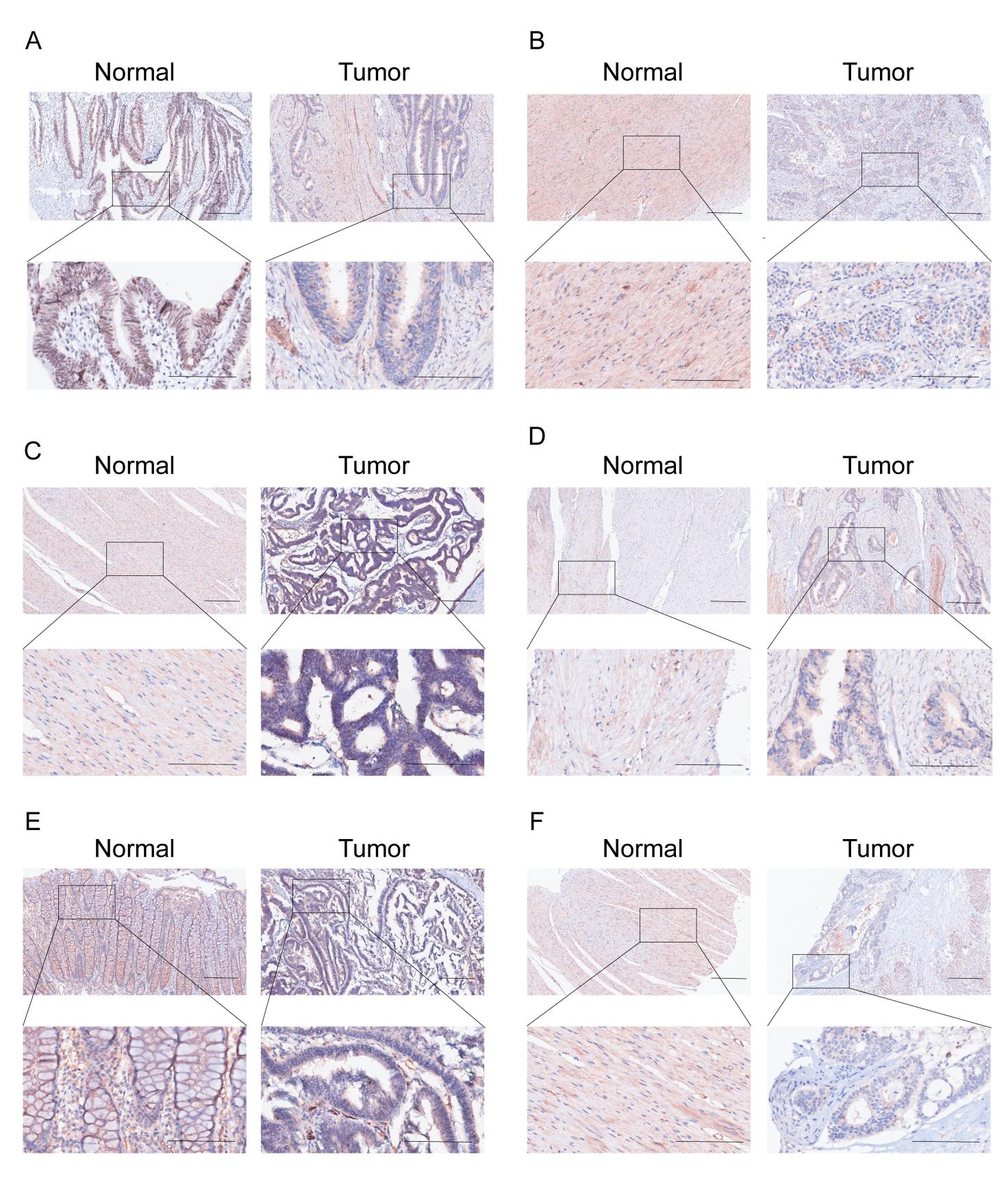


**Fig. S7. Representative immunohistochemical staining images of NRG1.**

(A-F) The strong staining of NRG1 in normal tissue and colorectal carcinoma tissue from 6 CRC patients. Bar,50 um
